# CD44‐Targeting Hydroxyapatite Nanoparticles (HAP) Induce Mitochondrial Dysfunction‐Driven PANoptosis and Immunogenic Cell Death (ICD) via Ca Overload in Colorectal Cancer

**DOI:** 10.1002/advs.75559

**Published:** 2026-05-06

**Authors:** Yao Xiao, Yuxuan Yang, Haosen Qiu, Jia Yang, Hao Liang, Zhaojun Jiang, Xiao Tong, Yongying Li, Qunfeng Huang, Jiaming Wu, Tian Lin, Jiang Yu, Min Liang

**Affiliations:** ^1^ Department of Oncology Department of Oncology GuangDong Engineering Technology Research Center of Biological Targeting Diagnosis, Therapy and Rehabilitation The Fifth Affiliated Hospital Guangzhou Medical University Guangzhou China; ^2^ Department of General Surgery Nanfang Hospital Southern Medical University Guangzhou China; ^3^ Department of General Surgery Xiangyang Central Hospital Affiliated Hospital of Hubei University of Arts and Science Xiangyang China; ^4^ Department of Respiratory and Critical Care Medicine Key Laboratory of Biological Targeting Diagnosis Therapy and Rehabilitation of Guangdong Higher Education Institutes The Fifth Affiliated Hospital Guangzhou Medical University Guangzhou China; ^5^ The Fifth Clinical College of Guangzhou Medical University Guangzhou China; ^6^ Department of Gastrointestinal Surgery Affiliated Hospital of Jiaxing University Jiaxing China

**Keywords:** calcium overload, colorectal cancer, immunogenic cell death, immunotherapy, PANoptosis

## Abstract

Colorectal cancer (CRC) remains therapeutically challenging due to high metastasis, recurrence, and immunotherapy resistance driven by tumor microenvironment‐mediated immune evasion. Immunogenic cell death (ICD) offers a promising strategy to reshape the immune microenvironment, yet existing ICD inducers suffer from poor targeting efficiency and insufficient death signal release. Here, we constructed a calcium overload‐based smart nanosystem, HA‐HAP@CUR, to achieve highly efficient ICD induction via a triple‐effect mechanism: hyaluronic acid (HA)‐mediated CD44 targeting enables tumor‐selective accumulation; pH‐responsive hydroxyapatite (HAP) degradation releases Ca^2+^ in the acidic tumor microenvironment; and curcumin (CUR) amplifies intracellular calcium overload by promoting endoplasmic reticulum Ca^2+^ release, collectively establishing a positive feedback loop disrupting calcium homeostasis. Mechanistically, calcium overload induces mitochondrial membrane potential dissipation and sustained mPTP opening, triggering mitochondrial oxidative stress and energy metabolic disorders. This mitochondrial crisis concurrently activates caspase‐3, GSDMD, and RIPK1, synergistically initiating apoptosis, pyroptosis, and necroptosis, ultimately converging into PANoptosis with potent immunostimulatory potential. This strategy, encompassing targeted accumulation, calcium storm activation, and multi‐modal cell death synergy, provides a biologically precise approach to overcoming immunotherapy resistance in CRC.

## Abstract 2

A hyaluronic acid‐functionalized hydroxyapatite nanoplatform co‐loaded with curcumin (HA‐HAP@CUR) is engineered to trigger calcium overload and mitochondrial dysfunction in colorectal cancer cells, culminating in integrated PANoptosis encompassing the simultaneous activation of apoptosis, necroptosis, and pyroptosis. The resulting release of damage‐associated molecular patterns (DAMPs) drives dendritic cell maturation and T cell activation, reprogramming immunologically “cold” tumors into “hot” tumors.

## 1 Introduction

Colorectal cancer (CRC), ranked third in global cancer incidence, causes a considerable public health burden, with the latest statistics reporting 1.9 million new cases and over 900,000 deaths annually [1]. Since early‐stage CRC is often asymptomatic, most patients present with advanced tumors, which significantly contributes to the high mortality rate. Standard treatment modalities for CRC include surgery, chemotherapy, radiotherapy, and immunotherapy. Approximately 70% of patients qualify for radical resection; however, some of these patients develop metastatic CRC postoperatively due to undetectable micrometastatic lesions, compromising the curative potential of surgery [2]. Patients who are not operable due to recurrence or metastasis must rely on palliative systemic therapy. Traditional anti‐cancer drugs primarily exert their effects through direct cytotoxicity to curb or kill tumor cells, but the cell death induced by these drugs is non‐immunogenic. The dead tumor cells are cleared by the body in the early stages, without activating the body's natural immunity [3, 4]. Tumor immunotherapy involves modulating the immune system to reactivate the “tumor immunity” cycle, restore and enhance anti‐tumor response, and improve immune cell recognition and cytotoxicity, aiming to restrain and eliminate tumor cells [5]. The KEYNOTE‐016 study, published in 2015, established microsatellite instability‐high (MSI‐H) or mismatch repair‐deficient (dMMR) as predictive biomarkers for immunotherapy response in CRC, thereby opening up a whole new world for CRC treatment [6]. With the rapid development of tumor biology, immunology, and genomics research, immunotherapy has achieved significant breakthroughs in the treatment of malignant tumors and is now considered the fourth major therapeutic modality alongside surgery, radiotherapy, and chemotherapy. Furthermore, the CheckMate 142 study has also demonstrated the survival benefits of immunotherapy in treating CRC [7]. Therefore, in the new guideline, immunotherapy is recommended upfront as a first‐line treatment for patients with metastatic CRC [8]. Although immunotherapy has demonstrated satisfactory efficacy in patients with MSI‐H or dMMR tumors, these subtypes account for less than 10–15% of CRC cases. The majority of patients have microsatellite stable (MSS) or mismatch repair proficient (pMMR) tumors, and these patients respond poorly to immunotherapy, mainly because the tumor microenvironment (TME) lacks immune cell infiltration—commonly referred to as ‘cold’ tumors [9, 10]. Therefore, overcoming the limitations of immunotherapy in MSS or pMMR CRC and transforming ‘cold’ tumors into ‘hot’ ones is a major scientific challenge in current cancer immunotherapy.

Immunogenic cell death (ICD) represents a distinct cell death modality. ICD reshapes the tumor immune microenvironment by the release of a large amount of damage‐associated molecular patterns (DAMPs) and induces the activation of antigen‐presenting cells (APCs). Notably, the exposure of calreticulin (CRT) on the cell membrane serves as an ‘eat‐me’ signal, facilitating phagocytosis by dendritic cells (DCs) and subsequently promoting their maturation. Adenosine triphosphate (ATP) releases a ‘find‐me’ signal to recruit phagocytes and promote the phagocytosis of damaged cells. Additionally, high mobility group box 1 (HMGB1) can bind to toll‐like receptor‐4 (TLR4), followed by activating the MyD88 signaling pathway. Subsequently, this activation leads to the induction of DC maturation, initiation of antigen‐specific T cell responses, and, ultimately, the activation of tumor‐specific T cell responses [11, 12, 13]. However, ICD induced by traditional chemotherapy faces inherent limitations in solid tumors: incomplete DAMPs release leads to disappointing immune activation efficiency, while a single mode of death is insufficient to achieve thorough tumor clearance [14, 15]. In recent years, the concept of PANoptosis has offered a new perspective for overcoming these bottlenecks. Unlike traditional programmed cell death modes, PANoptosis integrates the molecular components underlying apoptosis (Caspase cascade), pyroptosis (Gasdermin pores), and necroptosis (RIPK1/MLKL signaling) to form an irreversible cell death signaling network [16, 17]. Importantly, mitochondrial dysfunction (such as calcium overload‐induced decrease in mitochondrial membrane potential and ROS burst) can trigger apoptosis (cytochrome C), pyroptosis (NLRP3 inflammasome), and necroptosis (RIPK1/MLKL) signals, collectively forming a synergistic PANoptosis network [18, 19]. This process not only enhances the intensity of cell death but also amplifies ICD signals and reshapes the immune microenvironment through CRT membrane exposure, ATP secretion, HMGB1 release, and exocytosis [20]. However, precisely targeting and inducing the mitochondria‐PANoptosis‐ICD axis remains a challenge.

**SCHEME 1:**
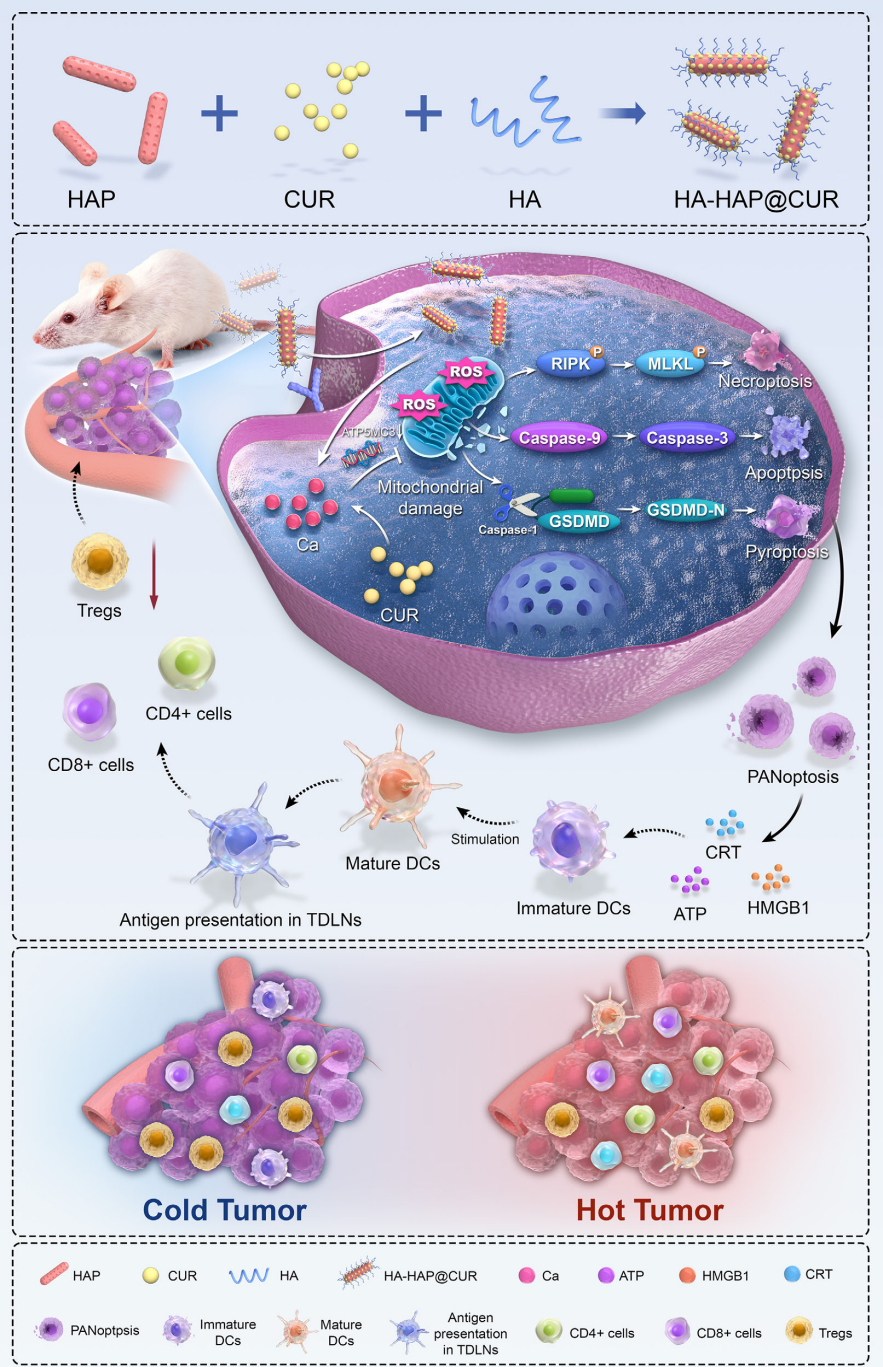
HA‐HAP@CUR nanoparticles drive self‐reinforcing antitumor immunity in CRC via a calcium overload/mitochondrial damage/PANoptosis axis and ICD‐mediated immune activation.

Studies have shown that Ca^2+^ is a crucial intracellular signaling molecule, and the extent of Ca^2+^ metabolic disorder in tumor cells is related to mitochondrial function. Notably, the regulation of calcium signal homeostasis plays a pivotal role in the transition between different modes of cell death [21]. When mitochondrial matrix calcium rises above the physiological levels, it induces the prolonged opening of the mitochondrial permeability transition pore (mPTP), which promotes the cytochrome c leakage, activates apoptotic pathways, and further amplifies the cell death signals through a calcium‐ROS positive feedback loop [22]. Previous studies from our group revealed that hydroxyapatite nanoparticles (HAP), as biocompatible nanocarriers, release high levels of Ca^2+^ in the acidic TME, causing mitochondrial damage and associated ICD [23, 24]. However, HAP has two major problems: insufficient tumor targeting ability and efflux of released Ca^2+^, which prevents sufficient ICD induction [25]. Studies have shown that CD44, highly expressed on the surface of CRC cells, is a hyaluronan receptor that can specifically recognize and bind to biomaterials with glycosaminoglycans as their basic structure, such as chondroitin sulfate and HA. Therefore, HA is expected to be used to increase the tumor targeting of HAP through surface modification [26]. Second, studies have shown that curcumin (CUR) can trigger the persistent release of Ca^2+^ from endoplasmic reticulum (ER) into cytoplasm in tumor cells [27]. Therefore, in this study, we loaded HA and CUR on the surface of HAP. As shown in the mechanism diagram, the HA‐HAP@CUR system achieves efficient ICD induction through a triple‐effect regulation mechanism. First, hyaluronic acid (HA)‐mediated CD44 targeting enabled nanoparticle accumulation at the tumor site. Second, pH‐responsive degradation of hydroxyapatite (HAP) in the acidic microenvironment released Ca^2+^. Finally, curcumin (CUR) amplified calcium signaling by promoting massive Ca^2+^ release from the ER into the cytoplasm, synergizing with the exogenous Ca^2+^ influx from HAP. These established a positive feedback loop that further disrupted calcium homeostasis. This vicious cycle ultimately triggers mitochondrial damage, activates PANoptosis, and releases DAMPs, thereby providing a novel therapeutic strategy for MSS and pMMR CRC (Scheme 1).

## 2 Materials and Methods

### 2.1 Chemicals

Nano‐hydroxyapatite powder (HAP, H875582), HA (H909936), and CUR (C805204) were purchased from Shanghai Macklin Biochemical Technology Co., Ltd. Polyethylene glycol (PEG‐NH_2_, MW: 10000, 98% purity) was obtained from Xi'an Ruixibio. N‐hydroxysuccinimide (NHS) and 1‐(3‐dimethylaminopropyl)‐3‐ethylcarbodiimide hydrochloride (EDC) were supplied by Aladdin Reagent (Shanghai). Phosphate‐buffered saline (PBS), fetal bovine serum (FBS), 0.05% trypsin, and Dulbecco's Modified Eagle Medium (DMEM) were purchased from Gibco (USA).

### 2.2 Cell Culture

Mouse Colon Carcinoma line (CT26 cell), obtained from the ATCC, was cultured in DMEM containing 10% FBS and antibiotics (penicillin 100 IU/mL, streptomycin 100 µg/mL) under standard conditions (37°C, 5% CO_2_). Once the cells reached approximately 80% confluence in the culture flasks, the cells were trypsinized and reseeded into dishes or plates for subsequent experiments.

### 2.3 Synthesis of HA‐HAP@CUR

First, HAP (25 mg) and PEG (125 mg, PEG‐NH_2_, MW: 10000, 98%) were dissolved in 50 mL of anhydrous ethanol. Next, a magnetic stirrer was used to continuously stir the mixture for 24 h at 60°C. The resulting mixture was washed several times sequentially with anhydrous ethanol and deionized water to obtain the PEG‐HAP‐NH_2_ NPs. To prepare the HA solution, HA (40 mg) was dissolved in 40 mL of deionized water by fully stirring to ensure complete dissolution. Then, EDC (160 mg) and NHS (23.4 mg) were added to the above HA solution with pH adjustment to 6–7 with hydrochloric acid, and the solution was stirred continuously for 3 h to activate the carboxyl group in HA by EDC to form an NHS‐stabilized intermediate. Subsequently, PEG‐HAP‐NH_2_ NPs (50 mg) were introduced and stirred at room temperature for 24 h. Then, the crude product was dialyzed (MWCO 3500 Da) against deionized water for 48 h to remove impurities, and then pre‐frozen at ‐80°C. Finally, HA‐HAP NPs were obtained by freeze‐drying. Next, CUR (5 mg) and HA‐HAP NPs (10 mg) were dissolved in 20 mL anhydrous ethanol and stirred for 24 h. The resulting nanoparticles were respectively washed with ethanol and Milli‐Q water, and separated by centrifugation (15 000 × *g*, 15 min). Finally, the product was kept in a fridge for next use.

### 2.4 Characterization of HA‐HAP@CUR

Surface morphology and size distribution of the synthesized products were characterized by TEM (JEM‐2100F, JEOL, Japan). Structural and elemental compositions were analyzed using XPS (Thermo Fisher Scientific, USA), FT‐IR (Nicolet IS5, Thermo Fisher Scientific), and UV–vis (UV‐2600, Shimadzu, Japan), which also enabled the determination of drug loading and encapsulation efficiency. The zeta potential and hydrodynamic diameter of the products were measured through DLS (Zetasizer Nano‐ZS, Malvern, UK). In addition, the in vitro release rate of synthesized products was tested using the dialysis method. Briefly, the NPs were dispersed in a small volume of PBS and transferred into a dialysis bag (MWCO: 3500 Da). The bag was then immersed in the release medium (PBS adjusted to pH 6.0, 6.8, and 7.4, respectively) and stirred at 50 rpm at 37°C. At the indicated time intervals, 1.0 mL of release medium was harvested and immediately replenished with an equal volume of fresh medium. The CUR concentrations within the release medium at different time intervals were determined by UV–vis, and the Ca^2+^ concentrations were quantified using ICP‐OES (ICP, AGILENT 725‐ES, USA). For cell morphology observation, CT26 cells were plated, treated with different formulations, subjected to pre‐fixation, post‐fixation, dehydration, drying, and metal coating before being examined with a scanning electron microscope (Hitachi S‐3000N, Japan) for image acquisition and data analysis.

### 2.5 Intracellular Uptake Assay

CT26 cells were seeded (1 × 10^5 cells per well) into laser confocal specialized dishes and incubated at 37°C for 24 h, followed by treatment with either Rhod‐labeled HA‐HAP NPs or HAP NPs. After 0, 2, 4, and 8 h of incubation, the cells were rinsed with PBS three times and fixed in 4% (w/v) paraformaldehyde (PFA) for 15 min at room temperature. The fixed cells were stained with 4’,6 diamidino‐2‐phenylindole (DAPI) (Solarbio, C0065) for 10 min. Following staining, the cells were washed twice with PBS, and fluorescence images were taken by a laser scanning confocal microscope (CLSM, LSM800, Zeiss).

### 2.6 CD44 Knockdown Assay

CT26 cells were transfected with CD44‐specific siRNA (si‐CD44) or negative control siRNA (si‐NC) using Lipofectamine 3000 (Invitrogen, L3000015) according to the manufacturer's instructions. At 48 h post‐transfection, cells were treated with Rhodamine‐labeled HA‐HAP@CUR NPs for 8 h. Intracellular fluorescence intensity was visualized using CLSM and quantified to evaluate nanoparticle uptake.

### 2.7 In Vitro Cytotoxicity and Anti‐Proliferation Assays

The cytotoxicity of the synthesized NPs on CT26 cells was evaluated using the Cell Counting Kit‐8 (CCK‐8) (DOJINDO, CK04) and EdU proliferation assay (RIBOBIO, C00053). For viability and proliferation tests, CT26 cells were cultured in 96‐well plates and pre‐incubated for 12 h, followed by 24 h incubation with varying concentrations of synthesized NPs. Then, the CCK‐8 reagent was added, and the absorbance (OD values) at 450 nm was measured using a microplate reader. For the EdU assay, cells were seeded in 24‐well plates for 12 h pre‐incubation. Next, different NPs were administered to treat cells for 24 h, and an EdU kit was utilized to detect cell proliferation. Apoptotic changes were visualized by YO‐PRO‐1/PI staining (MCE, HY‐K1092) after 24 h treatment of NPs; cells were incubated with YO‐PRO‐1 (1 µm) and PI (5 µg/mL) for 15 min and imaged using confocal microscopy (excitation: 488 nm for YO‐PRO‐1, 561 nm for PI).

### 2.8 Calcium Chelation Rescue Assay

CT26 cells were pre‐treated with 10 µM BAPTA‐AM (an intracellular calcium chelator) for 2 h, followed by treatment with HA‐HAP@CUR NPs for another 24 h. Cell viability was then evaluated using the CCK‐8 assay, as described previously. For PANoptosis biomarker detection, Western blot analysis was conducted to measure the cleavage of Caspase‐3, phosphorylation of MLKL, and N‐terminal cleavage of GSDMD, with GAPDH as the loading control.

### 2.9 Cell Death Pathway Inhibition Assay

CT26 cells were pre‐treated for 2 h with individual inhibitors (Z‐VAD‐FMK for apoptosis, Necrostatin‐1 for necroptosis, NLRP3/AIM2‐IN‐3 for pyroptosis) or a triple inhibitor cocktail (ZNN: Z‐VAD‐FMK + Necrostatin‐1 + NLRP3/AIM2‐IN‐3), prior to a 24‐h incubation with HA‐HAP@CUR NPs. Cell viability was evaluated using the CCK‐8 assay.

### 2.10 Assessment of Mitochondrial Function and ICD Markers

CT26 cells were treated with HAP NPs, HA‐HAP NPs, HAP@CUR NPs, or HA‐HAP@CUR NPs for 24 h, respectively. Mitochondrial permeability was assessed using an mPTP assay kit (Bestbio, BB‐48122), membrane potential was evaluated with a JC‐10 fluorescent probe (Bestbio, BB‐41052), and mitochondrial ROS production was detected with a MitoSOX Red fluorescent probe (Bestbio, BB‐44115), strictly following the manufacturer's protocols. To detect intracellular Ca^2+^ levels, a Fluo‐4 AM calcium fluorescent probe (Solarbio, F8501) was added, and fluorescence images were captured using CLSM. For the analysis of mitochondrial damage and ICD markers, treated cells were fixed with 4% PFA for 10 min, permeabilized with 0.1% Triton X‐100 for 15 min, and blocked with 5% BSA in PBS for 1 h at room temperature (25°C). The cells were then incubated with rabbit anti‐8‐OHdG (Bioss, bs‐1278R), rabbit anti‐HMGB1 antibody (Proteintech, 10829‐1‐AP), or rabbit anti‐CRT antibody (Proteintech, 10292‐1‐AP) at 4°C overnight. Subsequently, cells were washed with PBS, and Alexa Fluor 488‐conjugated goat anti‐rabbit IgG (bs‐0295G‐AF488) was applied for 2 h at room temperature in the dark. After washing with PBS, the cells were stained with DAPI for 10 min at 25°C, and the images were acquired using CLSM.

### 2.11 Mitochondrial ROS Source Verification

CT26 cells were seeded on laser confocal specialized dishes (1 × 10^5 cells per dish) and pre‐treated with 10 µm MitoTEMPO (a mitochondria‐targeted superoxide scavenger) for 2 h, then treated with HA‐HAP@CUR NPs for 24 h. For total intracellular ROS detection, cells were incubated with DCFH‐DA (Solarbio, CA1410) for 30 min. For mitochondrial superoxide detection, cells were incubated with MitoSOX Red (Bestbio, BB‐44115) for 20 min. Fluorescence images were captured by a fluorescence microscope and quantified for mean fluorescence intensity.

### 2.12 CD44 Expression Analysis in CRC Cell Lines

Normal colonic epithelial cells (HCEC‐1CT) and CRC cell lines (HT29, HCT116, CT26, MC38) were cultured to 80% confluence, then lysed with RIPA lysis buffer. Western blot analysis was performed to detect CD44 protein expression, with GAPDH as the internal control. The primary antibody anti‐CD44 (Proteintech,15675‐1‐AP) was used for detection.

### 2.13 Western Blot Analysis

Total proteins from different treatment groups of cells were extracted on ice using RIPA lysis buffer (Beyotime Biotechnology, P0013B) supplemented with a protease (MCE, HY‐K0010) and phosphatase (MCE, HY‐K0022) inhibitor cocktail. Equal amounts of protein (20 µg per lane) were separated by SDS‐PAGE and transferred onto PVDF membranes. After blocking with 5% bovine serum albumin (BSA) in PBST for 1 h at room temperature, the membranes were incubated overnight at 4°C with specific primary antibodies. To systematically detect the protein expression, the primary antibodies were grouped by manufacturers as follows: (1) Antibodies purchased from Affinity Biosciences: anti‐NLRP3 (DF7438), anti‐caspase‐1 (AF5418), anti‐cleaved‐caspase‐1 (AF4022), and anti‐GSDMD‐NT (DF13758); (2) Antibodies purchased from Bioss: anti‐caspase‐3 (bs‐0081R) and anti‐caspase‐9 (bs‐0049R), which were used to detect both their full‐length and cleaved forms, along with anti‐p‐MLKL (bsm‐54104R), anti‐MLKL (bs‐55131R), anti‐IL‐1β (bs‐0812R), and anti‐IFN‐γ (bs‐0480R); (3) Antibodies purchased from Proteintech: anti‐p‐RIPK‐1 (66854‐1‐Ig), anti‐RIPK‐1 (17519‐1‐AP), anti‐IL‐18 (10663‐1‐AP) and anti‐TNF‐α (17590‐1‐AP). An anti‐GAPDH antibody was used as the internal loading control. Following PBST washes, the membranes were incubated with HRP‐conjugated goat anti‐rabbit IgG (Beyotime, K1223) or goat anti‐mouse IgG (Beyotime, S0001) for 2 h at room temperature. Finally, the protein bands were visualized using an enhanced chemiluminescence (ECL) substrate (Meilunbio, MA0186) under a chemiluminescence imaging system (Bio‐Rad ChemDoc). The band intensities were quantified using ImageJ software.

### 2.14 In Vivo Anti‐Tumor Effects of HA‐HAP@CUR

A subcutaneous CT26 tumor model was established in 4‐6‐week‐old female BALB/c mice, with all procedures approved by the Animal Ethics Committee of Nanfang Hospital, Southern Medical University (Approval No. NFYY‐2021‐0157) and in line with ethical guidelines. For the single‐agent study: 1×10^7 CT26 cells were subcutaneously injected into the right hind flank of each mouse. After 12 days of tumor growth, mice were randomly divided into 5 groups (*n* = 3 per group) and tail‐vein injected with saline, HAP NPs, HA‐HAP NPs, HAP@CUR NPs or HA‐HAP@CUR NPs every 3 days for 3 doses. For anti‐PD‐1 combination study: After 7 days of tumor inoculation, mice were randomly divided into 4 groups (*n* = 3 per group) and tail‐vein injected with saline, anti‐PD‐1 antibody (100 µg per mouse), HA‐HAP@CUR NPs (50% of single‐agent dose), or combination (HA‐HAP@CUR NPs followed by anti‐PD‐1 antibody 24 h later), every 3 days for 3 doses. Tumor volume [(length × width^2^)/2, mm^3^] was measured every 3 days from the first injection. Mice were euthanized on day 15 by cervical dislocation; tumors were harvested, weighed, and fixed in 4% paraformaldehyde for further analysis.

### 2.15 Serum IFN‐β Measurement

CT26 tumor‐bearing mice were treated with HA‐HAP@CUR NPs or saline as described in the section “In vivo anti‐tumor effects of HA‐HAP@CUR”. At the experimental endpoint, mice were anesthetized, and blood was collected via the orbital venous plexus. Serum was separated by centrifugation at 3000 × *g* for 10 min. IFN‐β concentration was measured using a mouse IFN‐β ELISA kit (Solarbio, SEKM‐0032) according to the manufacturer's instructions.

### 2.16 Intratumoral Cytokine Measurement

Tumor tissues were harvested from the treated mice, weighed, and homogenized in ice‐cold RIPA lysis buffer containing protease inhibitors. The homogenates were centrifuged at 12 000 × *g* for 15 min at 4°C to collect supernatants. The concentrations of IFN‐γ (Solarbio, SEKM‐0031) and Granzyme B (Solarbio, SEKM‐0088) in the supernatants were determined using corresponding mouse ELISA kits following the manufacturer's protocols.

### 2.17 Hematoxylin‐Eosin Staining (H&E) and Immunohistochemistry (IHC) Analysis

Tumor tissues isolated from CT26‐bearing mice were processed for histology by fixation in 4% PFA, paraffin embedding, sectioning, and H&E staining for morphological evaluation. For IHC, sections were deparaffinized, underwent antigen retrieval and peroxidase blocking, followed by overnight incubation with primary antibodies (CRT and HMGB1). After washing, secondary antibodies (HRP‐conjugated anti‐rabbit IgG) were applied, and signals were visualized using DAB chromogen for 10 min at 25°C incubation and counterstained with hematoxylin.

### 2.18 In Vivo Analysis of Recruiting Immune Cells

Tumor tissues and spleens were isolated from the subcutaneous tumor models (including single‐agent and combination therapy groups) and digested into single‐cell suspensions for flow cytometry. Cells were first blocked with anti‐mouse CD16/CD32 (BD Biosciences, 553142) for 15 min at 4°C and then labeled with eFluor 506 viability dye (eBioscience, 65‐0866‐18) to exclude dead cells. Subsequently, surface markers were stained using APC anti‐CD3 (Abcam, ab239287), PE/Cy7 Anti‐CD45 (Abcam, ab210186), PerCP/Cy5.5 anti‐CD8 (Abcam, ab210329), FITC anti‐CD4 (Abcam, ab269349), and BV421 anti‐CD25 (BioLegend, 356113). To assess the populations of CD4^+^CD25^+^FOXP3^+^ regulatory T cell (Treg), the surface‐stained cells were fixed and permeabilized using a Foxp3/Transcription Factor Staining Buffer Set (eBioscience, 00‐5523‐00), followed by staining with PE anti‐Foxp3 antibody (Abcam, ab210231). For dendritic cell (DC) maturation analysis, spleen samples were stained with APC anti‐CD86 (Abcam, ab218757), PE‐Cy7 anti‐CD11c (Abcam, ab210310), and PE anti‐CD80 (Abcam, ab93507). All flow cytometry data were acquired using a FACSVerse instrument (Becton Dickinson, USA) and analyzed with FlowJo software.

### 2.19 Bone Marrow‐Derived Dendritic Cell (BMDC) Isolation and Differentiation

Mice were sacrificed by cervical dislocation. Femurs and tibias were harvested under sterile conditions, and bone marrow cells (BMCs) were flushed with RPMI‐1640 medium. After centrifugation at 1500 rpm for 10 min, red blood cells were lysed using RBC lysis buffer at room temperature for 2 min. BMCs were washed once with RPMI‐1640 medium and resuspended in complete medium containing RPMI‐1640 supplemented with 10% FBS, 1% penicillin‐streptomycin, 10 ng/mL rmGM‐CSF, and 10 ng/mL rmIL‐4. Cells were seeded at 1 × 10^6 cells/mL in 6‐well plates and incubated at 37°C with 5% CO_2_. The medium was refreshed on day 3, and BMDCs were collected on day 5 for subsequent experiments.

### 2.20 BMDC Co‐Culture and Flow Cytometry

Conditioned medium (CM) was collected from control, HHC‐treated, and HHC + ZNN‐treated tumor cells (after 24 h of drug treatment). BMDCs were seeded at 1 × 10^6 cells per well in 6‐well plates and co‐cultured with these CMs for 24 h. After co‐culture, cells were collected, centrifuged at 1500 rpm for 10 min, and the supernatant was discarded. Cells were then stained with antibodies against CD45, CD11c, CD86, and CD80, and analyzed by flow cytometry.

### 2.21 Statistical Analysis

All experiments were independently repeated at least three times, and data are presented as the mean ± standard deviation (SD). Statistical differences between multiple groups were evaluated using one‐way or two‐way analysis of variance (ANOVA) followed by Tukey's or Bonferroni's post‐hoc test. All statistical analyses were performed using the software SPSS 22.0. A *p*‐value of <0.05 was considered statistically significant.

## 3 Results and Discussion

### 3.1 Synthesis and Characterization of HA‐HAP@CUR

**FIGURE 1:**
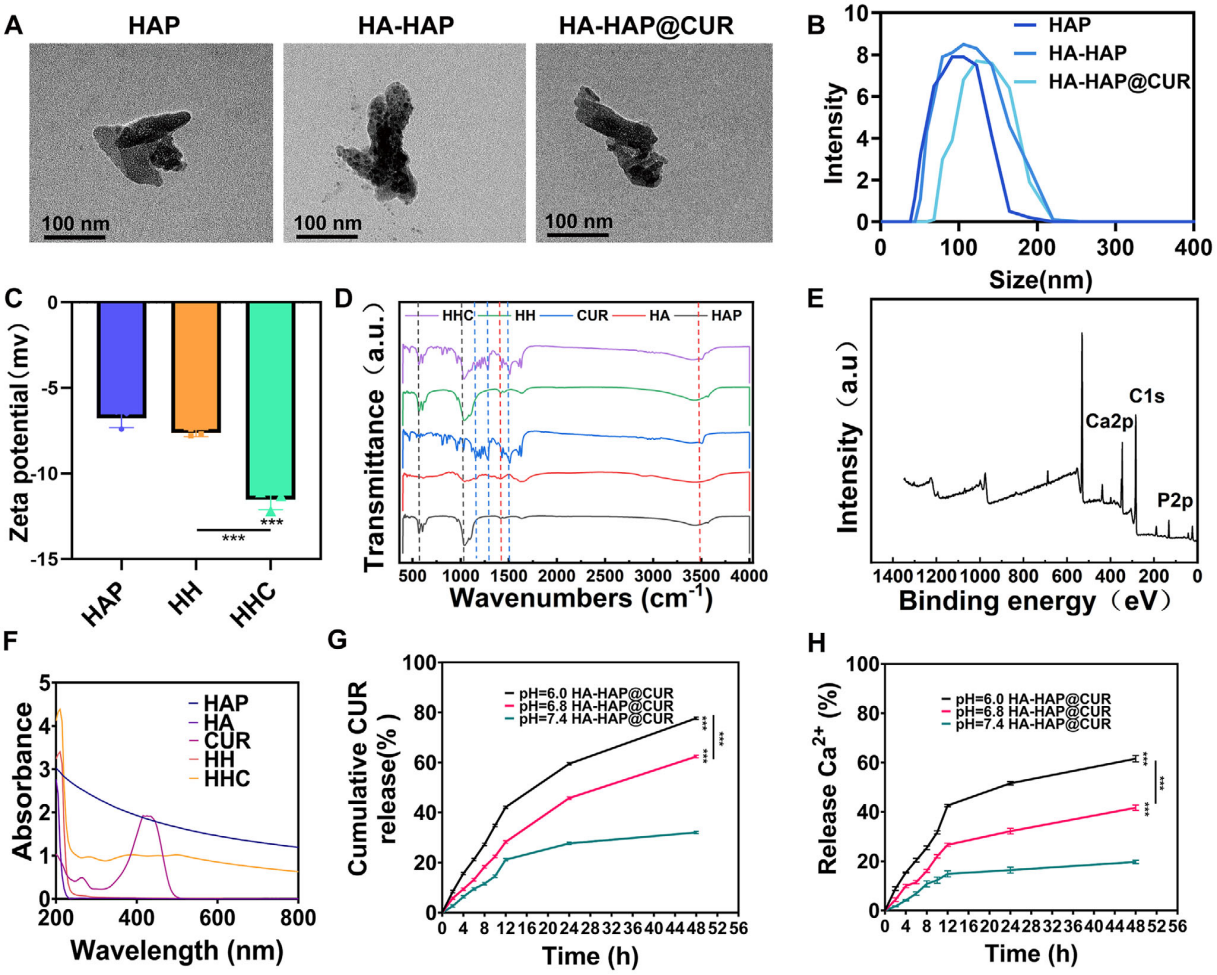
Characterization and Drug Release Properties of HA‐HAP@CUR Nanoparticles (A) TEM images of HAP NPs, HA‐HAP NPs, and HA‐HAP@CUR NPs, scale bar = 100 nm. (B) Particle size distribution curves of the three nanoparticles. (C) Zeta potential measurements of HAP NPs, HA‐HAP NPs, and HA‐HAP@CUR NPs (*n* = 3). (D) FTIR spectra of HAP NPs, HA, CUR, HA‐HAP NPs, and HA‐HAP@CUR NPs. (E) XPS analysis showing binding energy peaks of C1s, Ca2p, and P2p. (F) UV–vis absorption spectra of HAP NPs, HA‐HAP NPs, CUR, and HA‐HAP@CUR NPs. (G) Cumulative release curves of curcumin from HA‐HAP@CUR NPs under different pH conditions (6.0, 6.8, and 7.4) (*n* = 3). (H) Release percentage of Ca^2+^ ions under different pH conditions (6.0, 6.8, and 7.4) (*n* = 3). Data are presented as mean ± SD, ^*^p < 0.05, ^**^p < 0.01, ^***^p < 0.001.

It is observed by TEM images and Dynamic light scattering (DLS) results that HAP NPs, HA‐HAP NPs, and HA‐HAP@CUR NPs present rod‐like structures with a length of about 100 nm (Figure 1A,B). Measured zeta potential values are −7.2 mV, −8.1 mV, and −12.3 mV for HAP NPs, HA‐HAP NPs, and HA‐HAP@CUR NPs, respectively, denoting a modest shift in surface charge upon drug incorporation (Figure 1C). For FT‐IR spectroscopic results, specific characteristic peaks of HAP at 1037.58 cm^−1^ and 565.62 cm^−1^ within the range of 500–1100 cm^−1^ were observed, which corroborated the previous findings [28]. Furthermore, in the HA‐HAP NPs spectra, HA displayed the stretching vibrational peaks of O─H at 3425 cm^−1^ and carbonyl C═O at 1417 cm^−1^. Interestingly, the N─H bending vibrational peak (amide II band) is observed at 1633 cm^−1^, which is considered to be the presence of the amide bond formed by the connection between HAP and HA. In comparison to the HA‐HAP NPs group, the HA‐HAP@CUR NPs exhibited benzene ring, C─O, and C─O─C stretching vibration peaks of CUR at 1511 cm^−1^, 1281 cm^−1^, and 1157 cm^−1^, respectively, indicating successful loading of CUR (Figure 1D). XPS analysis confirmed the existence of C, P, and Ca elements, with the Ca2p peak at 346.05 eV, the C1s peak at 284.45 eV, and the P2p peak at 134.21 eV (Figure 1E). The UV spectra of HA‐HAP@CUR NPs exhibited distinct peaks at 200–210 nm and 425 nm, corresponding to HA and CUR, respectively (Figure 1F). Overall, these findings confirm the successful fabrication of HA‐HAP@CUR NPs. Previous studies have shown that HAP can significantly dissolve its deposit layer when exposed to acidic environments, indicating its biodegradability under low pH conditions (e.g., TME, typically having weakly acidic extracellular environments with a pH range from 6.0 to 6.8) [29]. Leveraging this property, we investigated the pH‐sensitive drug release profiles of CUR and Ca^2+^ in vitro. At a neutral pH of 7.4, only about 30% of CUR and 3% of Ca^2+^ were released over 48 h. In contrast, at pH 6.8, up to 55% of CUR and 30% of Ca^2+^ were discharged. Furthermore, at pH 6.0, nearly 63% CUR and 60% of Ca^2+^ are released. These findings suggest that HA‐HAP@CUR NPs may exhibit enhanced drug release rates in the TME (Figure 1G,H). Conventional nanocarriers (e.g., mesoporous silica), due to their structural inertness, often encounter the challenge of insufficient intracellular drug release, which limits drug bioavailability [30]. In contrast, the HAP nanocarrier employed in this study exhibits a unique pH‐triggered dissolution property. Upon stimulation by the acidic tumor microenvironment (TME), the rapid degradation of the HAP core releases the loaded CUR, achieving efficient intracellular drug delivery. More importantly, this degradation process converts the carrier, originally serving as a structural scaffold, into therapeutically active exogenous Ca^2+^. Consequently, our design elevates passive drug delivery to an integrated “delivery‐and‐therapy” strategy: Ca^2+^ not only acts as a signal of carrier disassembly but also synergizes with CUR to exert potent anti‐tumor effects. This dual‐function mechanism fundamentally overcomes the limitation of inert carriers functioning merely as “passive containers”, thereby maximizing the therapeutic potential and efficacy of the delivery system.

### 3.2 In Vitro Cellular Uptake and Cytotoxicity of HA‐HAP@CUR

**FIGURE 2:**
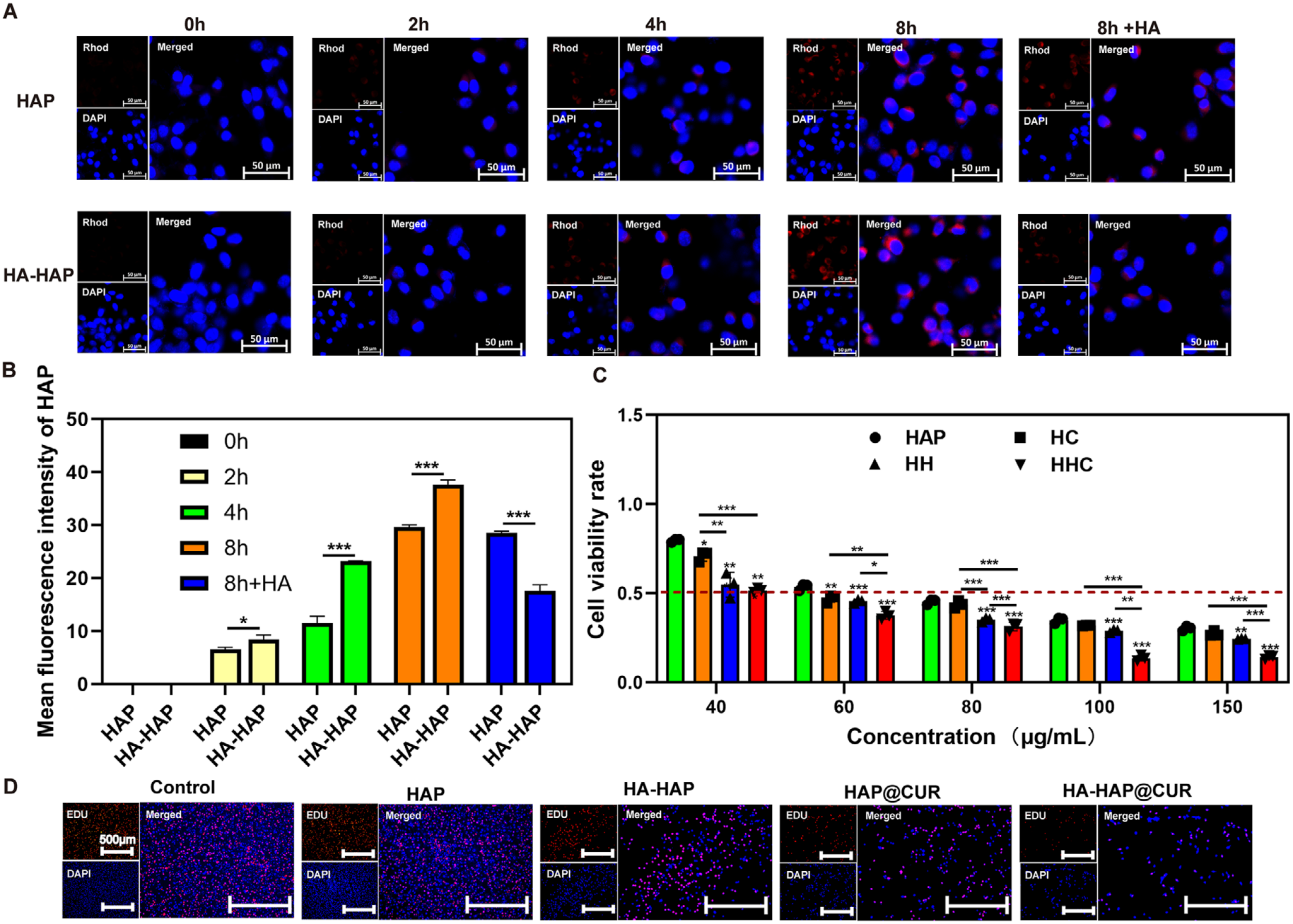
Nanoparticle uptake and cytotoxicity (A) Representative fluorescence microscopy images showing the time‐dependent cellular uptake (0 h, 2 h, 4 h, and 8 h) of HAP and HA‐HAP nanoparticles, as well as the competitive inhibition assay pre‐treated with free HA (8 h + HA). Red fluorescence indicates Rhodamine‐labeled nanoparticles, and blue fluorescence marks DAPI‐stained cell nuclei. Scale bar = 50 µm. (B) Quantitative analysis of the mean fluorescence intensity for the internalized HAP and HA‐HAP nanoparticles at corresponding time points and conditions. (C) Cell viability rates after treatment with different formulations (HAP, HC, HH, and HHC) at various concentrations (40 µg/mL, 60 µg/mL, 80 µg/mL, 100 µg/mL, and 150 µg/mL). (D) Representative EdU staining fluorescence images evaluating cell proliferation after treatment with Control, HAP, HA‐HAP, HAP@CUR, and HA‐HAP@CUR. Red fluorescence indicates EdU‐positive proliferating cells, and blue fluorescence represents DAPI‐stained nuclei. Scale bar = 500 µm. Data are presented as mean ± standard deviation, *n* = 3. ^*^ ***p*** < 0.05, ^**^ ***p*** < 0.01, ^***^ ***p*** < 0.001.

Nanomedicine delivery systems hold broad application prospects in the medical field due to their unique advantages, including desirable drug solubility, enhanced drug efficacy, and decreased side effects [31]. Hydroxyapatite (HAP) has been widely explored as a promising nanocarrier for anticancer applications. Nevertheless, the active targeting ability of HAP NPs is still insufficient, which is a crucial aspect requiring further optimization [32]. CD44 is a complex transmembrane adhesion glycoprotein that mediates lymphocyte homing and participates in cell‐cell adhesion. Moreover, its overexpression is closely associated with tumor drug resistance, recurrence, and metastasis [33]. We found that CD44 protein was significantly highly expressed in colorectal cancer cell lines (Figure S1), highlighting its potential as a robust target for CRC‐specific drug delivery. Utilizing this receptor‐ligand interaction, we modified our nanoparticles with HA to enhance their targeting capability via receptor‐mediated endocytosis, thereby improving their uptake. HA, a known modifier that specifically targets the CD44 receptor on CRC cells, was selected as the targeting modifier for HAP NPs, aiming to enhance selective cancer cell targeting. In our study, cellular uptake was assessed by incubating CRC cells with Rhodamine‐labeled HAP NPs or HA‐HAP NPs for up to 8 h, followed by tracking the fluorescence intensity of Rhod using CLSM. CLSM images showed a significant increase in the fluorescence intensity of HA‐HAP NPs at 2 h, 4 h, and 8 h compared to HAP. Concurrently, pretreatment with free HA significantly reduced the uptake of HA‐HAP NPs (Figure 2A,B). To further validate the specific role of CD44 in nanoparticle internalization, we employed RNA interference (RNAi) to knock down CD44 expression in CT26 cells. The results demonstrated that the uptake efficiency of HA‐HAP NPs was significantly decreased in CD44‐knockdown cells compared to control cells, with a fluorescence intensity reduction of approximately 20% (Figure S2). This directly confirmed that the CD44 receptor is a key molecule mediating the specific internalization of HA‐HAP NPs. In summary, both the competitive inhibition assay and the gene knockdown experiment jointly confirmed that HA modification significantly enhanced the targeted delivery capability of our nano system.

To evaluate the cytotoxicity of the developed NPs, CT26 cells were exposed to increasing concentrations of HAP NPs, HAP@CUR NPs, HA‐HAP NPs, or HA‐HAP@CUR NPs. The cell viability was measured using the CCK‐8 assay, and the results demonstrate that cell survival rates decreased significantly in all groups as the concentration increased (Figure 2C). Notably, HA‐HAP@CUR NPs exhibited the strongest cytotoxic effect in treatment, with an IC50 of approximately 40 µg/mL. We further evaluated the effect of the drugs on cell proliferation. The results show that the HA‐HAP@CUR NPs treatment group had the lowest proportion of EDU‐positive cells (24%) compared to the control group (50%), HAP group (45%), HAP@CUR NPs group (38%), and HA‐HAP NPs group (30%) (Figure 2D; Figure S3). Collectively, these findings suggest that HA‐HAP@CUR NPs exhibited significantly enhanced cytotoxicity, primarily driven by CD44‐targeted uptake, thereby warranting further mechanistic investigation. This enhanced therapeutic efficacy can be attributed to two synergistic factors: first, CD44‐mediated endocytosis promoted the intracellular accumulation of the nanodrugs; and second, the pH‐responsive dissolution of the HAP core in the acidic tumor microenvironment enabled efficient intracellular drug release.

### 3.3 Ca^2+^ Overload/Mitochondrial ROS Burst Positive Feedback Loop Induces Mitochondrial Functional Paralysis

**FIGURE 3:**
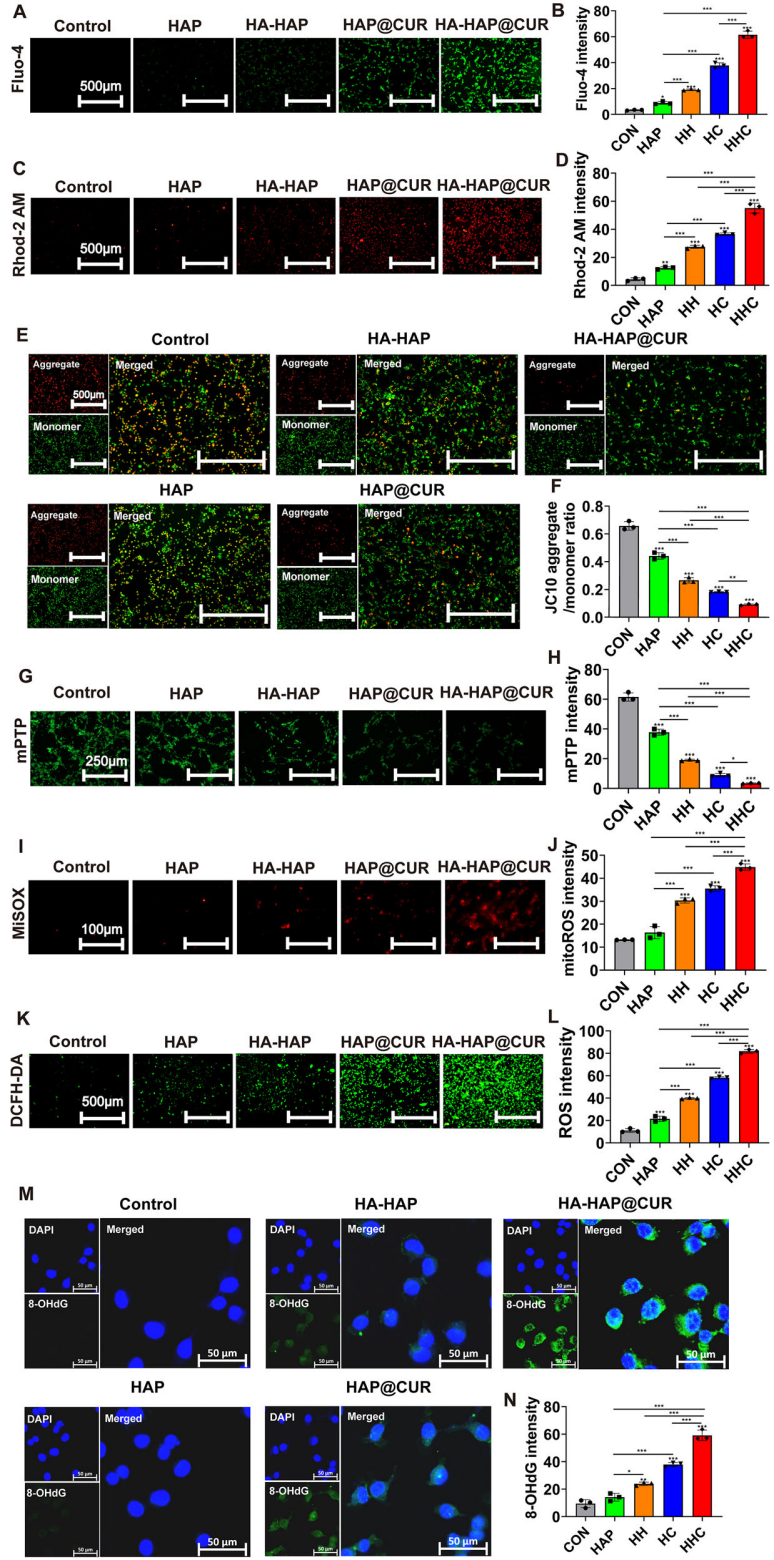
Effects of nanoparticles on cellular calcium ion levels, mitochondrial function, and oxidative stress. (A) Fluorescence images of intracellular Ca^2+^ levels after treatment with control, HAP NPs, HA‐HAP NPs, HAP@CUR NPs, and HA‐HAP@CUR NPs, scale bar = 500 µm. (B) Quantification of Fluo‐4 intensity reflecting cytosolic Ca^2^^+^ levels for each group. (C) Representative fluorescence images of cells treated with control, HAP NPs, HA‑HAP NPs, HAP@CUR NPs, and HA‑HAP@CUR NPs. Red signal indicates Rhod‑2 AM fluorescence (mitochondrial Ca^2+^), scale bar = 500 µm. (D) Quantification of mean Rhod‑2 AM fluorescence intensity for each group. (E) JC10 staining showing changes in mitochondrial membrane potential in each group, where red represents JC10 aggregates and green represents the monomeric form, scale bar = 500 µm. (F) Quantitative analysis of the JC10 aggregate/monomer ratio for each group. (G) mPTP opening status in cells of each group, scale bar = 250 µm. (H) mPTP opening assessed by fluorescence intensity. (I) MitoSOX red fluorescence staining showing mitochondrial superoxide levels, scale bar = 100 µm. (J) Quantitative analysis of MitoSOX fluorescence intensity for each group. (K) Representative fluorescence images of DCFH‐DA (green) in cells after treatment with Control, HAP NPs, HA‑HAP NPs, HAP@CUR NPs, and HA‑HAP@CUR NPs, indicating changes in total cellular ROS, scale bar = 500 µm. (L) Total intracellular ROS levels measured by DCFH‐DA fluorescence. (M) Fluorescence images of DAPI nuclear staining (blue) and 8‐OHdG staining (green) in cells from each group, scale bar = 50 µm. (N) Quantified 8‐OHdG fluorescence intensity indicating oxidative DNA damage. Data are presented as mean ± standard deviation. *n* = 3. ^*^ ***p*** < 0.05, ^**^ ***p*** < 0.01, ^***^ ***p*** < 0.001.

Based on the finding by Xia et al. that hydroxyapatite (HAP) can be biodegraded and release calcium ions in an acidic microenvironment [34], we employed the Fluo‐4 AM probe to monitor cytoplasmic Ca^2+^ levels in CT26 cells. The results show that negligible green fluorescence was detected in the control group, whereas weak signals appeared in cells treated with HAP NPs and HA‐HAP NPs, indicating calcium release from HAP. Notably, CUR‐loaded formulations (HAP@CUR NPs and HA‐HAP@CUR NPs groups) show a significant increase in intracellular Ca^2+^ levels (Figure 3A,B), indicating that CUR aggravates intracellular calcium overload and disrupts cellular homeostasis.

Mitochondria function as the central organelle for energy production and play a pivotal role in regulating intracellular calcium homeostasis. Persistent calcium overload can disrupt mitochondrial function, with key manifestations including collapse of energy metabolism and increased oxidative stress [35]. To determine whether the cytosolic Ca^2+^ overload extends to mitochondria, Rhod‐2 AM staining was performed to measure mitochondrial Ca^2+^. Fluorescence analysis shows that mitochondrial Ca^2+^ fluorescence was markedly increased in the CUR‐loaded HAP@CUR and HA‐HAP@CUR groups, with the HA‐HAP@CUR group exhibiting the strongest signal (Figure 3C,D), confirming mitochondrial calcium overload. To evaluate the mitochondrial response to calcium overload, mitochondrial membrane potential was assessed using JC‐10 staining, which demonstrates a significant reduction to 15% of control levels after 24 h HA‐HAP@CUR treatment (Figure 3E,F). To further elucidate the underlying mechanism, we examined the opening status of the mitochondrial permeability transition pore (mPTP). As shown in the figure, the Control group retained strong green fluorescence, whereas the green fluorescence of the HA‐HAP@CUR NPs group was significantly weakened, indicating sustained mPTP opening and loss of inner membrane integrity (Figure 3G,H). The collapse of mitochondrial membrane potential and sustained opening of mPTP are usually accompanied by excessive generation of mitochondrial reactive oxygen species (mitoROS). Detection using the mitochondria‐specific superoxide probe MitoSOX Red shows that the mitochondrial ROS level in the HA‐HAP@CUR treatment group was 3.5 times that of the control group (Figure 3I,J). To verify the specific origin of intracellular ROS and establish a direct causal link, we performed experiments with MitoTEMPO, a mitochondria‐targeted superoxide scavenger. Notably, this pretreatment effectively abolished HA‐HAP@CUR‐induced ROS generation, confirming mitochondria as the major source of this oxidative burst (Figures S4 and S5). To further confirm whether mitochondrial ROS contribute to increased cytosolic ROS, we measured total intracellular ROS using DCFH‐DA. The results revealed a pronounced rise in intracellular ROS in the HA‐HAP@CUR group that paralleled the increase in MitoSOX signal (Figure 3K,L), supporting that mitoROS are released into the cytosol and amplify cellular oxidative stress. This oxidative stress was not limited to mitochondria, but also led to oxidative damage of nuclear DNA. Moreover, 8‐OHdG immunofluorescence staining (Figure 3M,N) shows that the DNA oxidation damage level in the HA‐HAP@CUR NPs group increased by 5.5‐fold, further confirming the extensive cellular damage of mitochondrial‐derived ROS. These results demonstrate that cytoplasmic calcium overload induced by HA‐HAP@CUR nanoparticles further promotes the pathological accumulation of calcium ions within mitochondria. This process triggers the sustained opening of the mitochondrial permeability transition pore (mPTP) and alterations in the mitochondrial membrane potential, leading to a burst of mitochondrial reactive oxygen species (mitoROS) production. This is accompanied by the generation of large quantities of oxidatively damaged mitochondrial DNA (ox‐mitoDNA) and its subsequent release into the cytoplasm. The resulting mitochondria‐derived signals establish a positive feedback regulatory loop between the disruption of calcium homeostasis imbalance and severe mitochondrial oxidative stress. Ultimately, this cascading mechanism effectively converts localized ionic disturbances into global oxidative damage and irreversible mitochondrial functional dysfunction.

### 3.4 PANoptosis Execution and ICD Signal Amplification

**FIGURE 4:**
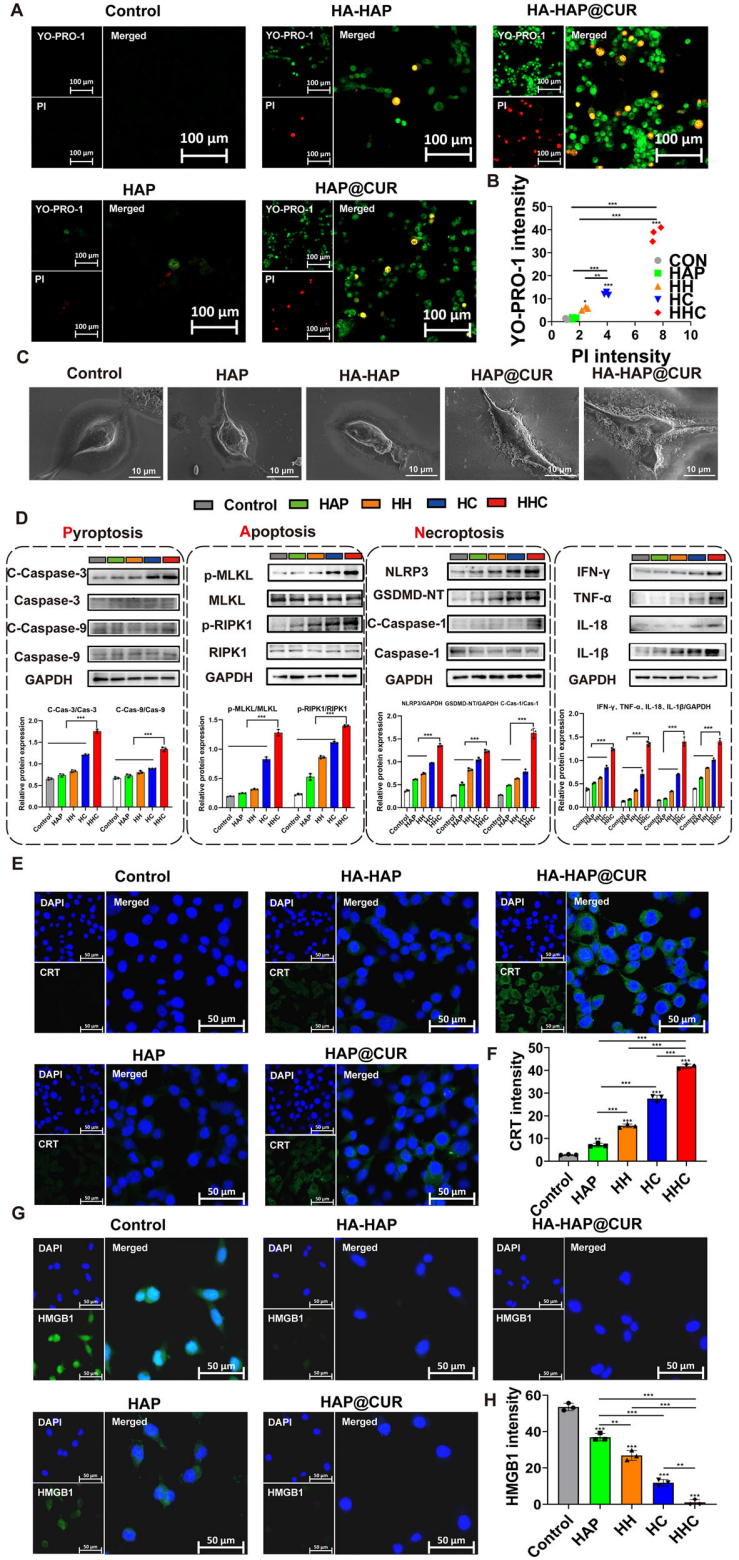
Nanoparticles induce cellular pan‐apoptosis (A) YO‐PRO‐1 (green)/PI (red) double fluorescent staining showing cell death in each group, scale bar = 100 µm. (B) Quantification of YO‐PRO‐1 and PI fluorescence intensity. (C) Microscopic images of cell morphology after treatment with control, HAP, HA‐HAP, HAP@CUR, and HA‐HAP@CUR, scale bar = 10 µm. (D) Western blot analysis of pyroptosis, apoptosis, and necrosis‐related protein expression in each experimental group. (E) Immunofluorescence staining of calreticulin (CRT) (green) and DAPI nuclear staining (blue), scale bar = 50 µm. (F) Quantitative analysis of CRT fluorescence intensity in each group. (G) Immunofluorescence staining of high mobility group box 1 (HMGB1) (green) and DAPI nuclear staining (blue), scale bar = 50 µm. (H) Quantitative analysis of HMGB1 fluorescence intensity in each group. Data are presented as mean ± standard deviation, *n* = 3. ^*^ ***p*** < 0.05, ^**^ ***p*** < 0.01, ^***^ ***p*** < 0.001.

Mitochondria, as central regulators of cell death, can trigger multiple death signaling pathways simultaneously when dysfunctional. To characterize cell death morphology, YO‐PRO‐1/PI double staining was performed using CLSM (Figure 4A,B). The results show that the HA‐HAP@CUR NPs treatment group exhibits three cell subpopulations: YO‐PRO‐1^+^/PI^−^, YO‐PRO‐1^+^/PI^+^, and YO‐PRO‐1^−^/PI^+^. The proportion of YO‐PRO‐1^+^/PI^+^ double‐positive cells is significantly higher in the HA‐HAP@CUR NPs group than in other groups. This phenotype cannot be explained by classical apoptosis theory, as it simultaneously exhibits altered membrane permeability (YO‐PRO‐1^+^) and loss of membrane integrity (PI^+^). Next, we assessed cell ultrastructure using scanning electron microscopy. The results show that cells in each group exhibit varying degrees of swelling, deformation, and membrane blebbing (Figure 4C), suggesting the synergistic action of pyroptosis‐related membrane pore formation (pyroptotic GSDMD pores) and necroptosis‐related membrane rupture (MLKL pores). These findings indicate that HA‐HAP@CUR‐treated cells may have undergone membrane‐rupture cell death driven by multiple pathways, consistent with the typical morphological features of PANoptosis. To functionally validate PANoptosis as an integrated program rather than independent pathways, we employed specific cell death inhibitors. While individual inhibition of apoptosis (Z‐VAD‐FMK), necroptosis (Necrostatin‐1), or pyroptosis (NLRP3/AIM2‐IN‐3) yielded only partial rescue of HA‐HAP@CUR‐induced cell death, reflecting compensatory mechanisms, the combined “ZNN” cocktail (targeting all three pathways) offered significantly superior protection, restoring viability nearly to control levels (Figure S6). This compelling evidence underscores the simultaneous activation and functional interdependence of these pathways, confirming HA‐HAP@CUR triggers an integrated PANoptosis network rather than merely co‐occurring, independent cell death. We further investigated whether HA‐HAP@CUR NPs triggered PANoptosis‐a novel form of programmed cell death involving the synergistic activation of apoptosis, necroptosis, and pyroptosis. Western blot analysis (Figure 4D) demonstrates simultaneous activation of marker proteins associated with all three cell death pathways following HA‐HAP@CUR NPs treatment, and the activation of the three pathways is highly synchronized, indicating their integration within a common signaling network rather than functioning independently. These results demonstrate that PANoptosis, as an integrated cell death network, is critical in mediating HA‐HAP@CUR‐induced cytotoxicity. To definitively establish calcium overload as an indispensable upstream initiator of PANoptosis, we conducted rescue experiments using the intracellular calcium chelator BAPTA‐AM. Pre‐treatment with BAPTA‐AM significantly mitigated HA‐HAP@CUR‐induced cytotoxicity, restoring cell viability from ∼57% to ∼76% (Figure S7). This direct attenuation of cell death by calcium chelation already suggested a crucial role for calcium. Furthermore, western blot analysis (Figure S8) demonstrated that BAPTA‐AM robustly suppressed HA‐HAP@CUR‐induced cleavage of Caspase‐3, phosphorylation of MLKL, and N‐terminal cleavage of GSDMD, key markers of apoptosis, necroptosis, and pyroptosis, respectively. These findings firmly establish intracellular calcium overload as a causal upstream trigger for the complex PANoptosis observed in HA‐HAP@CUR‐treated CRC cells, underscoring the critical role of calcium homeostasis in regulating this intricate cell death pathway. This mechanistic clarity is crucial, as it differentiates calcium overload as a causal driver rather than merely a secondary or correlative phenomenon, thereby solidifying the proposed mechanism by which HA‐HAP@CUR nanoparticles exert their antitumor effects by orchestrating the intricate cascade leading to PANoptosis. This multi‐pathway synergistic PANoptosis mechanism effectively overcomes the compensatory survival defects of traditional single‐mode treatments and facilitates the synergistic release of DAMPs through an integrated death signaling network. The expression of three major ICD markers was further examined in order to explore whether this multi‐modal cell death could enhance tumor immunogenicity. Immunofluorescence results show, after HA‐HAP@CUR NPs treatment, that the amount of calreticulin (CRT), which translocated from the endoplasmic reticulum to the cell membrane surface, increased 15‐fold (Figure 4E,F). HAP NPs, HA‐HAP NPs, and HAP@CUR NPs treatment groups show a gradual decrease in HMGB1 fluorescence intensity, with the most pronounced reduction observed in the HA‐HAP@CUR NPs group (Figure 4G,H). Moreover, a 2‐fold increase in ATP secretion is detected in the HA‐HAP@CUR NPs group (Figure S9). Evaluation of serum IFN‐β levels in tumor‐bearing mice provided functional evidence for HA‐HAP@CUR‐induced “true ICD” (Figure S10). Supernatant transfer assays combined with PANoptosis blockade further established that immune activation is a downstream consequence of PANoptosis, and PANoptosis inhibition impairs ICD signal release and subsequent DC maturation (Figures S11–S13). These findings demonstrate that HA‐HAP@CUR can trigger mitochondrial stress via calcium overload, ultimately activating PANoptosis. Compared to classical apoptosis for targeting chemoresistant tumors, the induction of PANoptosis offers unique mechanistic advantages. Unlike apoptosis, which is often tolerogenic and easily evaded via caspase mutations, the concurrent activation of pyroptosis and necroptosis physically ruptures the cell membrane. This catastrophic membrane damage is essential for the massive efflux of DAMPs, translating local intracellular stress into robust, systemic antitumor immunity.

### 3.5 In Vivo Anti‐Tumor Efficiency of HA‐HAP@CUR NPs

**FIGURE 5:**
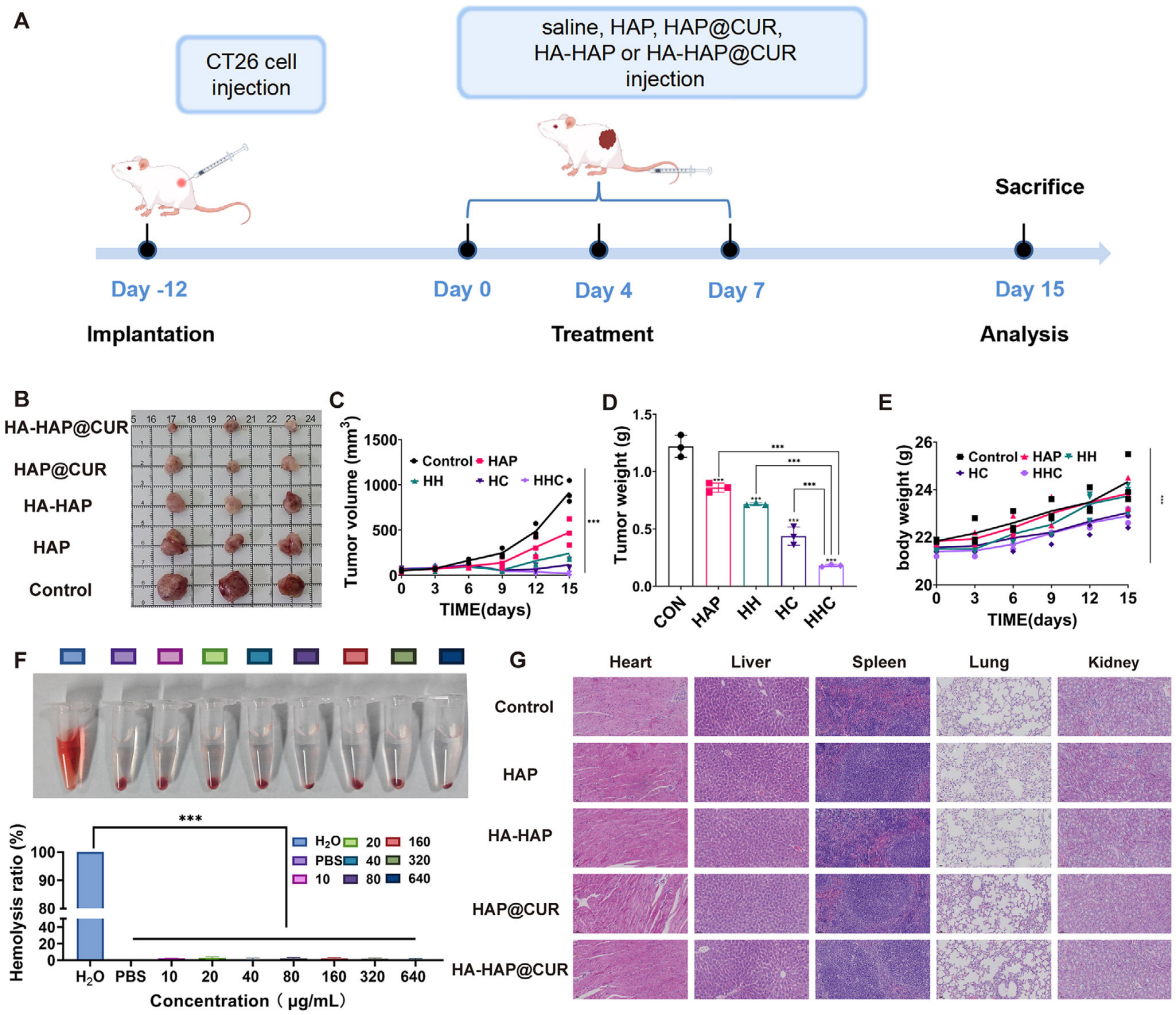
In vivo anti‐tumor effects and safety evaluation of nanoparticles (A) Schematic diagram of treatments. (B) Picture of subcutaneous tumors harvested from each treatment group (Control, HAP NPs, HA‐HAP NPs, HAP@CUR NPs, and HA‐HAP@CUR). (C) Time‐dependent changes in tumor volume over 15 days for each treatment group. (D) Comparison of tumor weights among different groups at the end of treatment. (E) Comparison of body weight among mice in different treatment groups over 15 days. (F) Hemolysis test and quantitative analysis of hemolysis rates at different nanoparticle concentrations (10‐640 µg/mL), with H_2_O and PBS as positive and negative controls, respectively. (G) Histopathological examination of major organs (heart, liver, spleen, lung, and kidney) from mice in different treatment groups using H&E staining. Data are presented as mean ± standard deviation, *n* = 3. ^*^ ***p*** < 0.05, ^**^ ***p*** < 0.01, ^***^ ***p*** < 0.001.

To evaluate the potential therapeutic effects of HA‐HAP@CUR NPs in vivo, a subcutaneous CT26 tumor mouse model was established, and mice were randomly divided into 5 groups: control group (physiological saline), HAP NPs, HA‐HAP NPs, HAP@CUR NPs, or HA‐HAP@CUR NPs. Mice were intravenously injected with the respective treatments and euthanized on day 15 (Figure 5A). Tumor growth is significantly inhibited in all nanoparticle‐treated groups compared with controls. Among these, HA‐HAP@CUR NPs exhibited the most potent inhibitory effect, with tumor volume reduced by 99.5% and tumor weight reduced by 93.6% compared to the control group. HAP@CUR NPs (41%) also demonstrated good anti‐tumor activity, while the anti‐tumor effects of HAP NPs and HA‐HAP NPs alone were relatively limited, indicating that CUR loading is crucial for enhancing the therapeutic efficacy. Furthermore, the tumor therapeutic effect of HA‐HAP@CUR NPs is superior to that of HAP@CUR NPs, confirming that HA modification enhances the accumulation of nanoparticles in the tumor site through CD44‐mediated targeting. These results clearly demonstrate that HA‐HAP@CUR NPs have a significant inhibitory effect on tumor growth (Figure 5B–D). We further evaluated the therapeutic potential of HA‐HAP@CUR NPs in combination with anti‐PD‐1 immune checkpoint blockade, and in vivo experiments validated their robust synergistic antitumor efficacy in CT26 tumor‐bearing mice (Figure S14). Systemic safety was evaluated through body weight monitoring, hemolysis testing, and histopathology. There was no significant difference in body weight among the groups throughout the treatment period, indicating good tolerance to the treatment (Figure 5E). Blood compatibility analysis (Figure 5F) shows that the positive control (H_2_O) induced complete hemolysis (98%), while the hemolysis rate in the full concentration range of 10–640 µg/mL shows no statistically significant difference compared to the PBS negative control. Histopathological examination of the major organs (heart, liver, spleen, lung, kidney) reveals no evidence of inflammatory infiltration or tissue damage (Figure 5G), further demonstrating the good biocompatibility of this nanoplatform. Taken together, these findings indicate that HA‐HAP@CUR nanoparticles combine potent antitumor activity with excellent biocompatibility, providing strong support for their clinical translation as a tumor‐targeted delivery system. Although metal‐ion‐based nanotherapeutics (such as iron, copper, zinc, and manganese) have shown potential in tumor catalytic therapy [36], their clinical translation has long been hampered by two core bottlenecks: first, the non‐degradability of exogenous heavy metal ions in vivo and the associated risks of long‐term accumulation; second, off‐target toxicity due to non‐specific ion distribution, which can induce systemic oxidative stress and inflammatory damage [37]. Addressing these limitations, the HA‐HAP@CUR nanoparticles constructed in this study achieve a unity of safety and efficacy through an endogenous ion interference strategy. On the one hand, the degradation products of the HAP carrier are intrinsic Ca^2+^, avoiding the non‐metabolizable nature and chronic toxicity risks associated with exogenous metals. On the other hand, HA‐mediated CD44 targeting precisely restricts the Ca^2+^ overload effect to tumor cells, minimizing non‐specific interference with normal tissues. Subacute toxicity assessment showed that HA‐HAP@CUR NPs achieved significant tumor inhibition without causing histopathological abnormalities in major organs. This tumor intervention strategy, based on calcium ion homeostasis, demonstrates a superior safety window and clinical translation prospect compared to exogenous conventional metal‐based nanotherapeutics.

### 3.6 HA@HAP‐CUR NPs Inhibited CRC Proliferation and Transformed the TME from Cold to Hot

**FIGURE 6:**
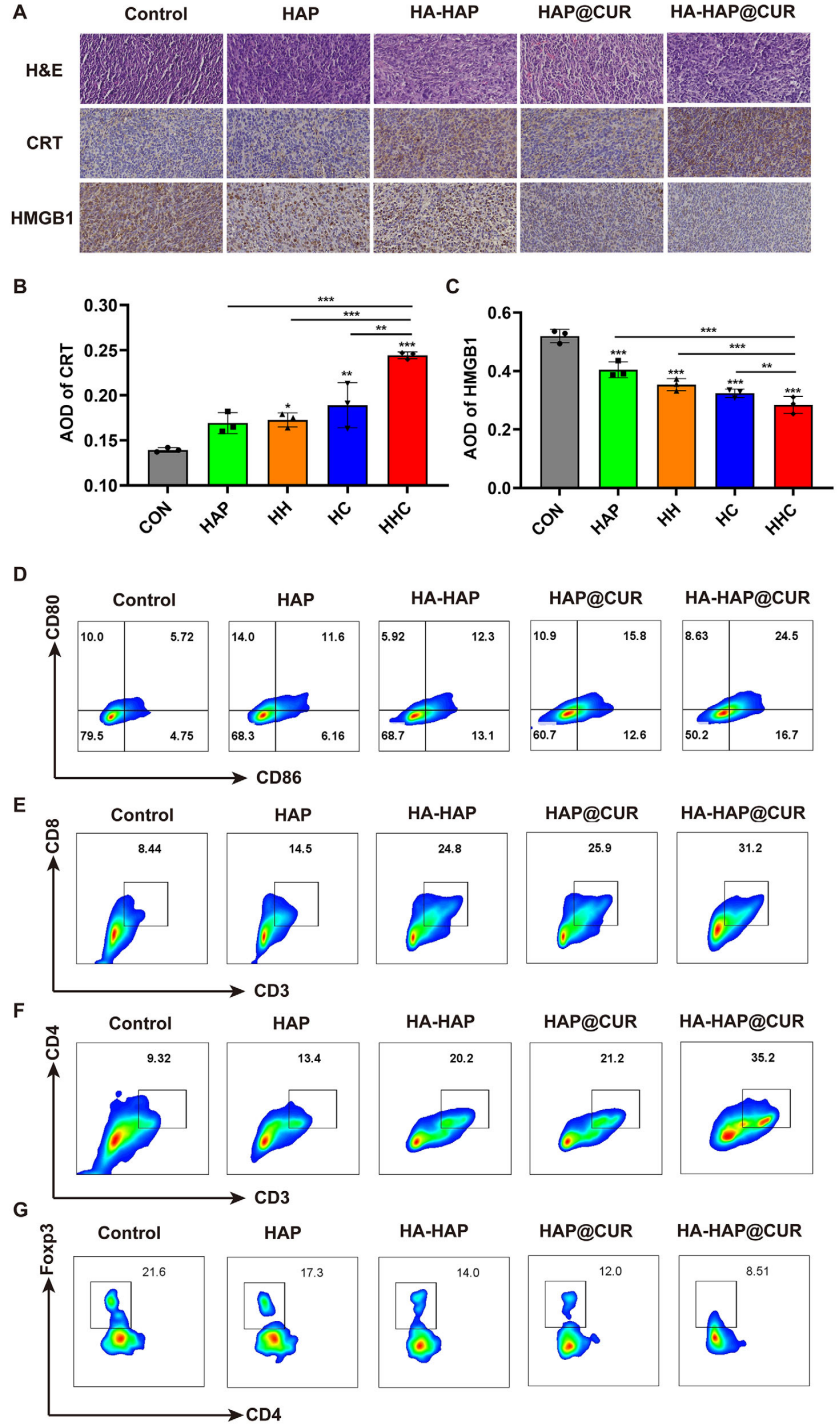
Effects of HA‐HAP@CUR NPs on ICD and TME in tumor tissue (A) H&E staining and immunohistochemical staining of CRT and HMGB1 in tumor tissues from different treatment groups (Control, HAP NPs, HA‐HAP NPs, HAP@CUR NPs, and HA‐HAP@CUR NPs). (B) Quantitative analysis of the average optical density (AOD) of CRT expression in tumor tissues from each group. (C) Quantitative analysis of the average optical density (AOD) of HMGB1 expression in tumor tissues from each group. (D) Flow cytometry analysis of dendritic cells (CD80/CD86) in tumor tissues from each group; numbers indicate the percentage of positive cells. (E) Flow cytometry analysis of CD3^+^CD8^+^ cells (cytotoxic T cells) in tumor tissues from each group. (F) Flow cytometry analysis of CD3^+^CD4^+^ cells (helper T cells) in tumor tissues from each group. (G) Flow cytometry analysis of CD4^+^Foxp3^+^ cells (regulatory T cells) in tumor tissues from each group. Data are presented as mean ± SD, *n* = 3. ^*^ ***p*** < 0.05, ^**^ ***p*** < 0.01, ^***^ ***p*** < 0.001.

To validate the therapeutic efficacy of nanoparticles in vivo, paraffin‐embedded tumor sections were analyzed for proliferation and immunogenic cell death (ICD) markers using H&E, CRT, and HMGB1 staining. Compared with controls, the HA‐HAP@CUR NPs treatment group exhibited a looser tumor cell arrangement with noticeable widening of intercellular spaces, confirming the significant tumor‐suppressing effect of HA‐HAP@CUR NPs. Concurrently, the surface exposure of the ICD marker CRT was markedly increased, while the intracellular HMGB1 levels were significantly reduced (Figure 6A), indicating its extracellular release and the robust induction of ICD. Growing evidence suggests that ICD enhances the immunogenicity of tumor cells and activates the host's innate immune response, thereby eliciting specific antitumor immunity [38]. DCs are central to coordinating immune responses and act as key antigen‐presenting cells bridging innate and adaptive immunity [39, 40]. To investigate the potential of HA‐HAP@CUR NPs in promoting DC maturation and enhancing immune responses, we conducted flow cytometric analysis of DC maturation markers CD80 and CD86 expression on DCs isolated from mouse spleens. The results present compelling evidence that HA‐HAP@CUR NPs treatment significantly upregulates the expression of CD80 and CD86 on DCs when compared with untreated controls (Figure 6D; Figure S15). This significant upregulation highlights the enhanced DC maturation triggered by HA‐HAP@CUR NPs, ultimately improving antigen‐presenting capacity and initiation of antitumor immunity. In anti‐tumor immunity, CD8^+^ T cells play a pivotal role by secreting perforin and granzyme B, thereby reinforcing cytotoxic T lymphocyte (CTL) responses [41]. In contrast, CD4^+^CD25^+^FOXP3^+^ regulatory T (Treg) cells impose suppressive effects on the immune system, weakening the effectiveness of tumor‐effector T cells, which frequently results in immune escape by tumors. Notably, elevated Treg cell counts in various malignancies are associated with poor prognosis [42]. The flow cytometric analysis reveals alterations in T cell populations in the tumor tissues from HA‐HAP@CUR NPs treatment group. Specifically, HA‐HAP@CUR NPs treatment results in markedly increased infiltration of CD8^+^ and CD4^+^ T cells in the TME (Figure 6E,F; Figures S16 and S17). Furthermore, the frequency of Treg cells is significantly reduced after HA‐HAP@CUR NPs treatment, suggesting attenuation of Treg‐mediated immunosuppression (Figure 6G; Figure S18). Collectively, our results demonstrate that HA‐HAP@CUR NPs not only inhibit tumor growth but also effectively remodel the TME by amplifying ICD signals, promoting DC maturation, and enhancing effector T‐cell responses, thereby establishing strong anti‐tumor immunity. To elucidate the mechanism underlying the observed synergy between HA‐HAP@CUR and anti‐PD‐1 therapy, we further analyzed the functional activation of tumor‐infiltrating T cells and TME remodeling after combination treatment. Subsequent analysis of intratumoral cytotoxic effector molecules further confirmed the functional activation of tumor‐infiltrating T cells in both HA‐HAP@CUR‐treated and HA‐HAP@CUR + anti‐PD‐1 combination‐treated mice (Figures S19 and S20). Building on this functional validation of T cell activity, comprehensive flow cytometry analysis elucidated the profound remodeling of the tumor immune microenvironment induced by the combination therapy (Figure S21). CRC, particularly the mismatch‐repair proficient (pMMR) subtype, is notoriously refractory to immune checkpoint blockade (ICB) due to insufficient T cell infiltration [43]. The significant shift in the CD8^+^ T cell‐to‐Treg ratio observed here confirms the successful conversion of an immunosuppressive “cold” TME into an inflamed “hot” phenotype. By acting as a potent immunogenic trigger through calcium‐driven PANoptosis, HA‐HAP@CUR robustly primes the immune system.

## 4 Conclusions

This study focuses on ICD and constructs a smart nano‐system, HA‐HAP@CUR NPs, based on calcium overload. This system achieves efficient ICD induction through a triple‐fold regulatory mechanism. First, HA‐mediated CD44 targeting enabled nanoparticle accumulation at the tumor site. Second, pH‐responsive degradation of HAP in the acidic microenvironment released Ca^2+^. Finally, CUR amplified calcium signaling via promoting Ca^2+^ release from the endoplasmic reticulum into the cytoplasm. These established a positive feedback loop that further disrupted calcium homeostasis. The resultant persistent cytosolic calcium overload initially triggers mitochondrial membrane potential dissipation and sustained opening of the mitochondrial permeability transition pore (mPTP), driving pathological mitochondrial calcium influx and subsequent severe mitochondrial oxidative stress. This state of mitochondrial crisis serves as an upstream signaling hub that concurrently activates key effector proteins, including caspase‐3, GSDMD, and RIPK1, to orchestrate the coordination and crosstalk among apoptosis, pyroptosis, and necroptosis, which ultimately defines a PANoptosis phenotype with potent immunostimulatory potential. This process leads to the release of immunogenic molecules (CRT, HMGB1, ATP), promotes DC maturation, and activates CD8^+^ T cells (directly killing tumor cells) and CD4^+^ T cells (secreting immune factors). This multi‐modal strategy—combining targeted delivery, PANoptosis induction, and immune activation—effectively enhances the host immune response, resulting in efficient and durable anti‐tumor effects.

## Funding

This work was supported by grants from Graduate Research Project of Guangzhou Municipal Education Bureau (2024312340), Xiangyang Central Hospital Youth Talent Development Program (2025RCYC‐013), Guangzhou Medical University Innovation ability improvement plan project (02‐408‐240603131121), Guangzhou Major Medical Disciplines Project (2025‐2027), Guangzhou Municipal Health Science and Technology General Guidance Project (20261A010109), National Oncology Clinical Key Speciality (2023‐GJZK‐001), Technology Program of Zhejiang Province (2025KY348), and Jiaxing Key Discipline of Traditional Chinese Medicine Surgery (2023‐ZYYCX‐002).

## Ethics Approval and Consent to Participate

All the animal experiments were conducted in accordance with the guidelines and the ethical standards of Nanfang Hospital Animal Ethics Committee.

## Consent for Publication

All authors agree to be published.

## Conflicts of Interest

The authors declare no conflicts of interest.

## Supporting information




**Supporting File**: advs75559‐sup‐0001‐SuppMat.docx.

## Data Availability

The data that support the findings of this study are available from the corresponding author upon reasonable request.
