# Supplementary material for: CD44‐Targeting Hydroxyapatite Nanoparticles (HAP) Induce Mitochondrial Dysfunction‐Driven PANoptosis and Immunogenic Cell Death (ICD) via Ca Overload in Colorectal Cancer
Source: Adv Sci (Weinh). 2026 May 6;13(42):e75559. doi: 10.1002/advs.75559 (PMC13335995; doi:10.1002/advs.75559)
Supplement: Supplementary file 1 — Supporting File: advs75559‐sup‐0001‐SuppMat.docx. [file ADVS-13-e75559-s001.docx]

**CD44-targeting hydroxyapatite nanoparticles (HAP) induce mitochondrial dysfunction-driven PANoptosis and immunogenic cell death (ICD) via Ca overload in colorectal cancer**

***Supplementary files***


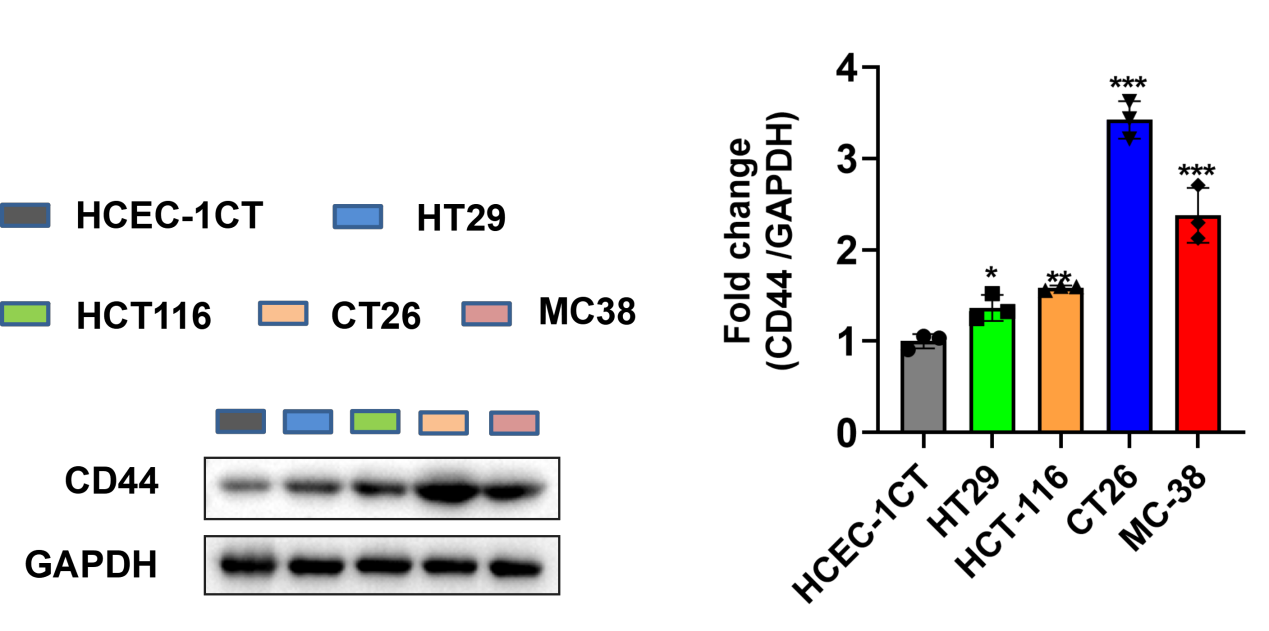


Figure S1. Expression levels of the CD44 receptor in normal colonic epithelial cells (HCEC-1CT) and a panel of colorectal cancer (CRC) cell lines (HT29, HCT116, CT26, MC38). Data are presented as mean ± SD. ******p*** < 0.05, *******p*** < 0.01, ********p*** < 0.001.


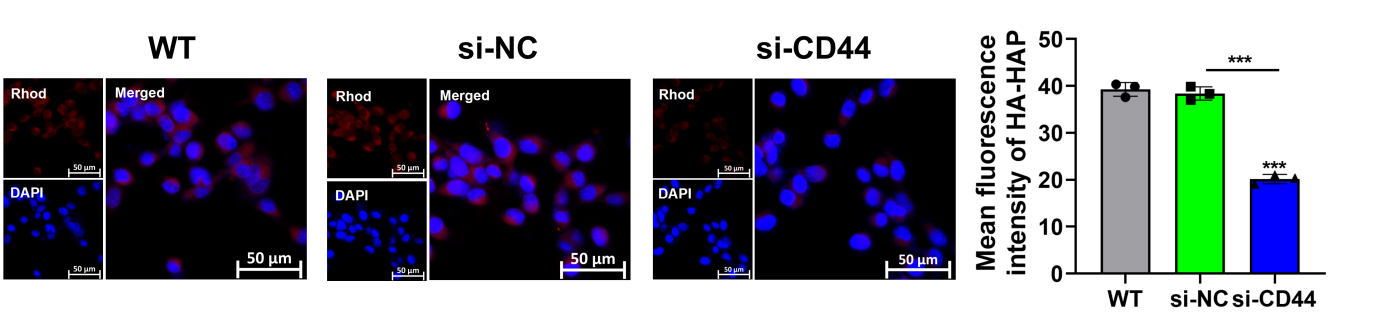


Figure S2. Representative fluorescence microscopy images (left) and corresponding quantitative analysis of the mean fluorescence intensity (right) showing the cellular internalization of Rhodamine-labeled HA-HAP nanoparticles in wild-type (WT) cells, cells transfected with negative control siRNA (si-NC), and CD44-knockdown cells (si-CD44) after 8 h of incubation. Red fluorescence indicates the internalized nanoparticles, and blue fluorescence represents DAPI-stained cell nuclei. Scale bars = 50 μm. Data are presented as mean ± SD (n = 3). ******p*** < 0.001.

**
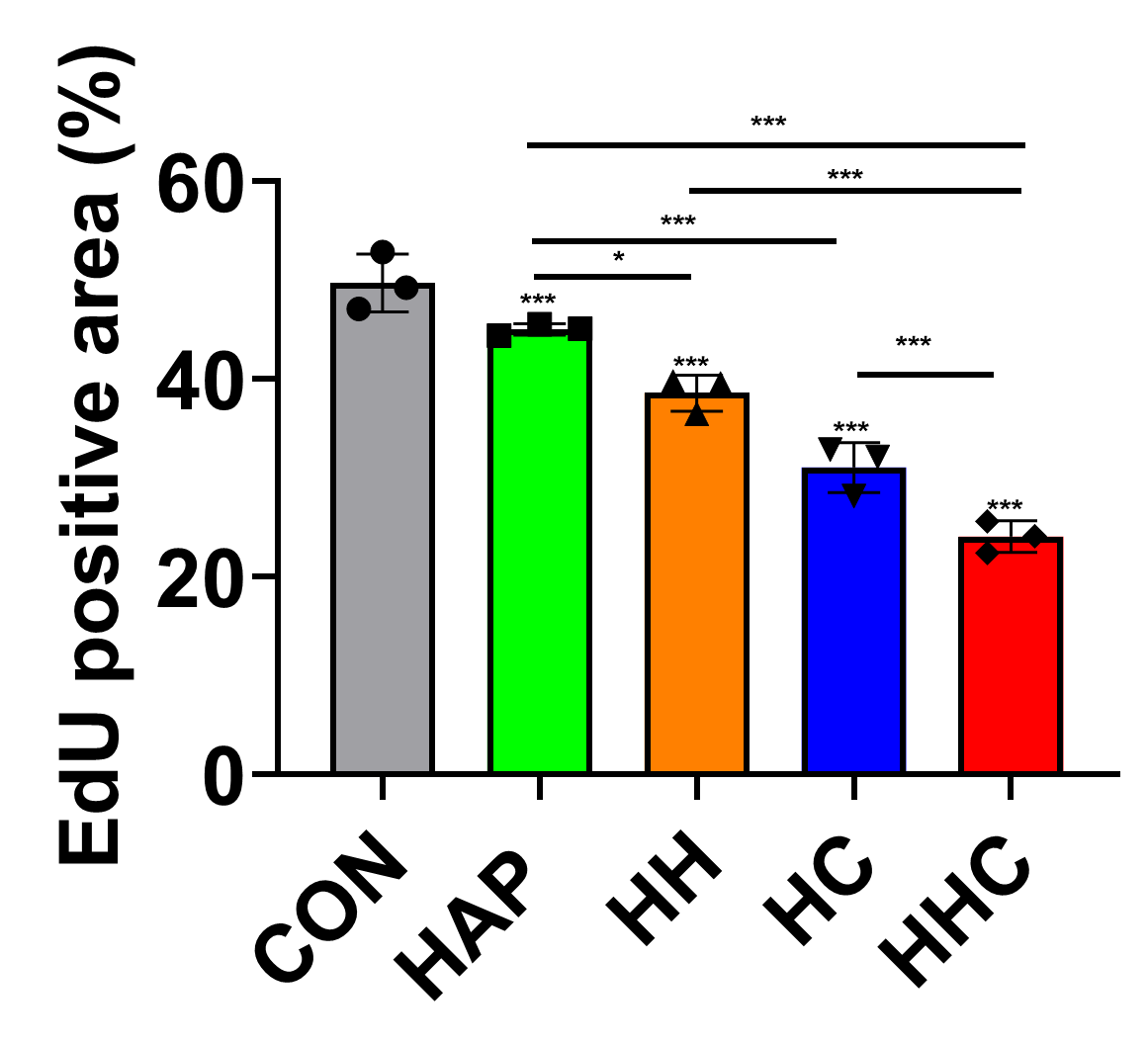
**

Figure S3. Quantification of cell proliferation based on EdU-positive area. Data are presented as mean ± SD, n = 3. ******p*** < 0.05, *******p*** < 0.01, ********p*** < 0.001.


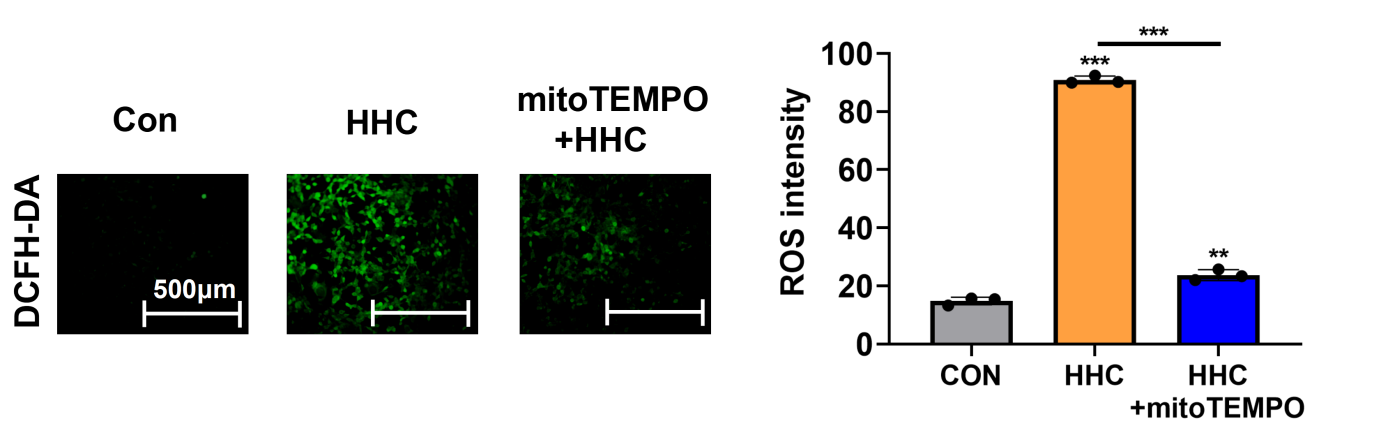


Figure S4. Representative fluorescence images and quantitative analysis of total intracellular ROS (detected using DCFH-DA, green) in CT26 cells treated with HHC in the presence or absence of the mitochondria-targeted superoxide scavenger MitoTEMPO. Data are presented as mean ± SD. *******p*** < 0.01, ********p*** < 0.001.


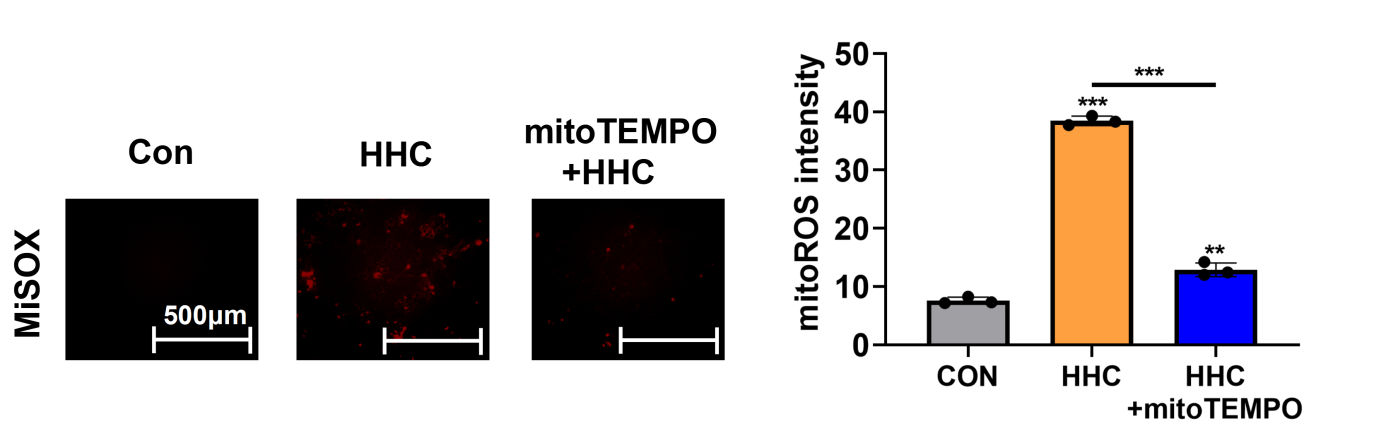


Figure S5. Representative fluorescence images and quantitative analysis of mitochondrial superoxide (detected using MitoSOX, red) in CT26 cells treated with HHC in the presence or absence of the mitochondria-targeted superoxide scavenger MitoTEMPO. Data are presented as mean ± SD. *******p*** < 0.01, ********p*** < 0.001.


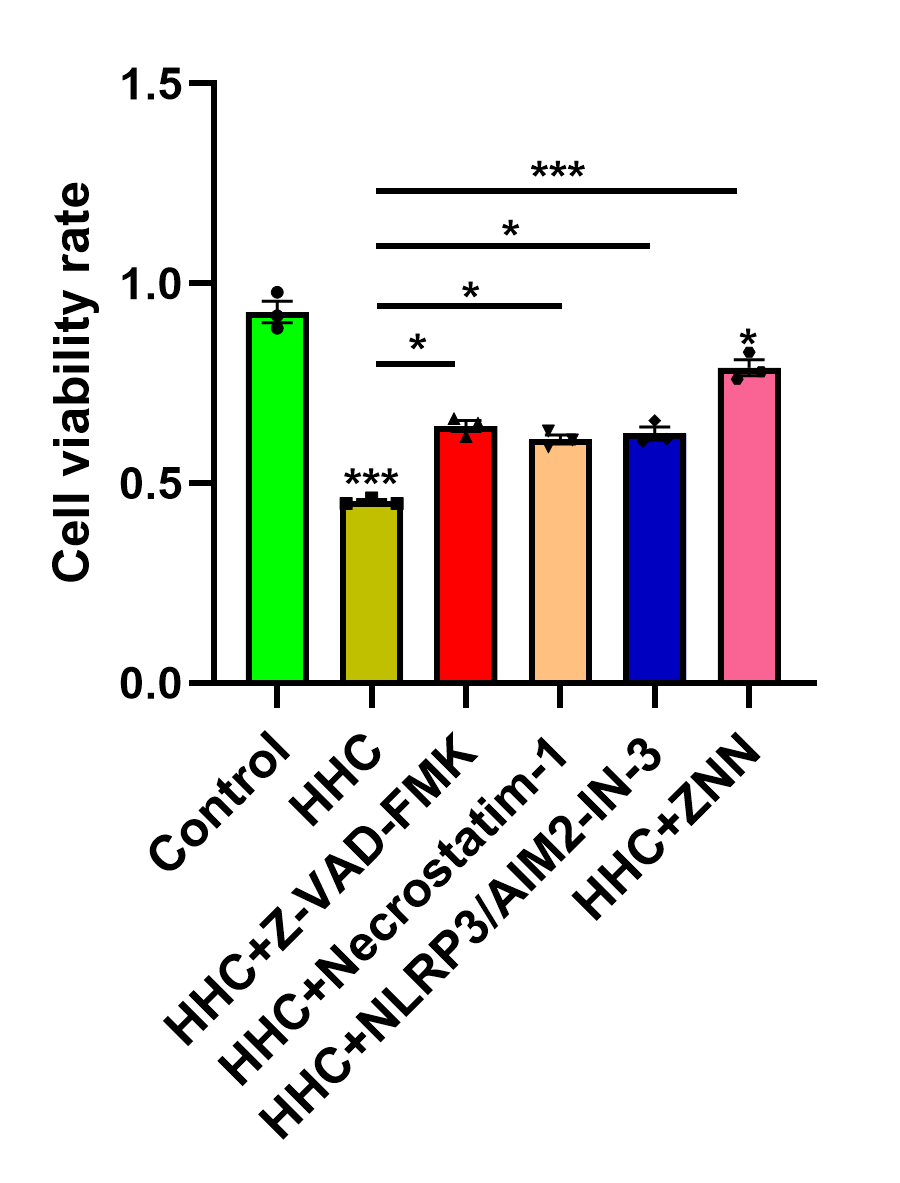


Figure S6. Relative cell viability of CT26 cells treated with HHC in the presence of individual cell death inhibitors (Z-VAD-FMK for apoptosis, Necrostatin-1 for necroptosis, or NLRP3/AIM2-IN-3 for pyroptosis) or a combined inhibitor cocktail. “ZNN” refers to a triple inhibitor cocktail comprising Z-VAD-FMK, Necrostatin-1, and NLRP3/AIM2-IN-3 to comprehensively block PANoptosis. Data are presented as mean ± SD (n = 3). ******p*** < 0.05, *******p*** < 0.01, ********p*** < 0.001.


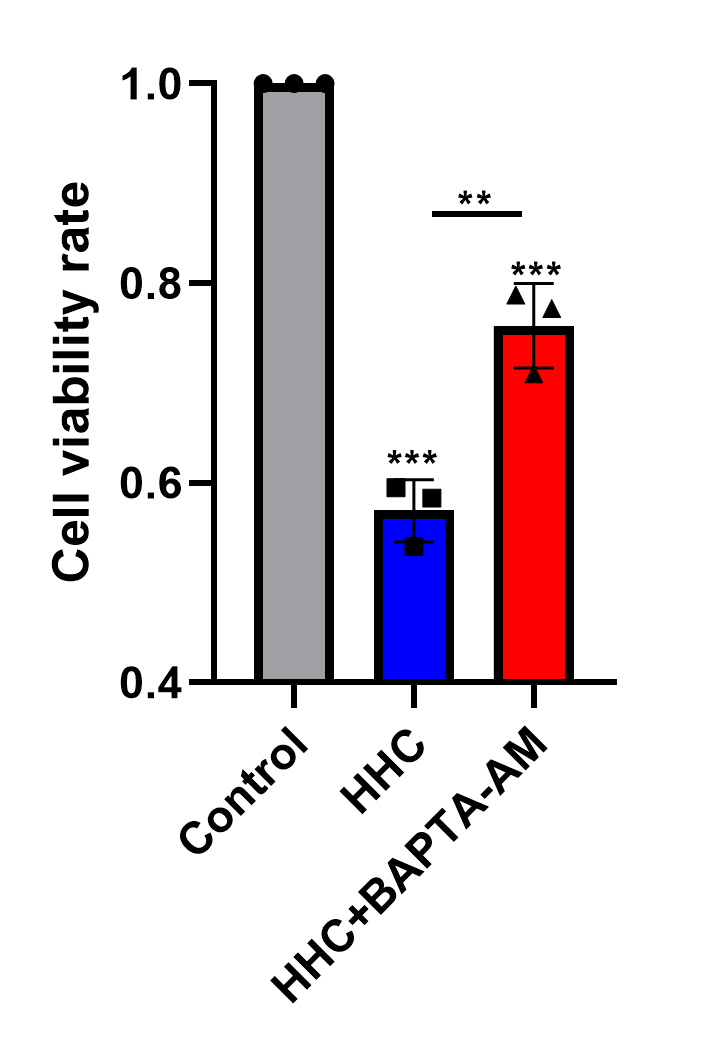


Figure S7. Cell viability, as assessed by CCK-8 assay, in cells treated with PBS (Control), HHC, or HHC following pretreatment with the intracellular calcium chelator BAPTA-AM. Data are presented as mean ± SD (n=3). *******p*** *<* 0.01*, *******p*** *<* 0.001*.*


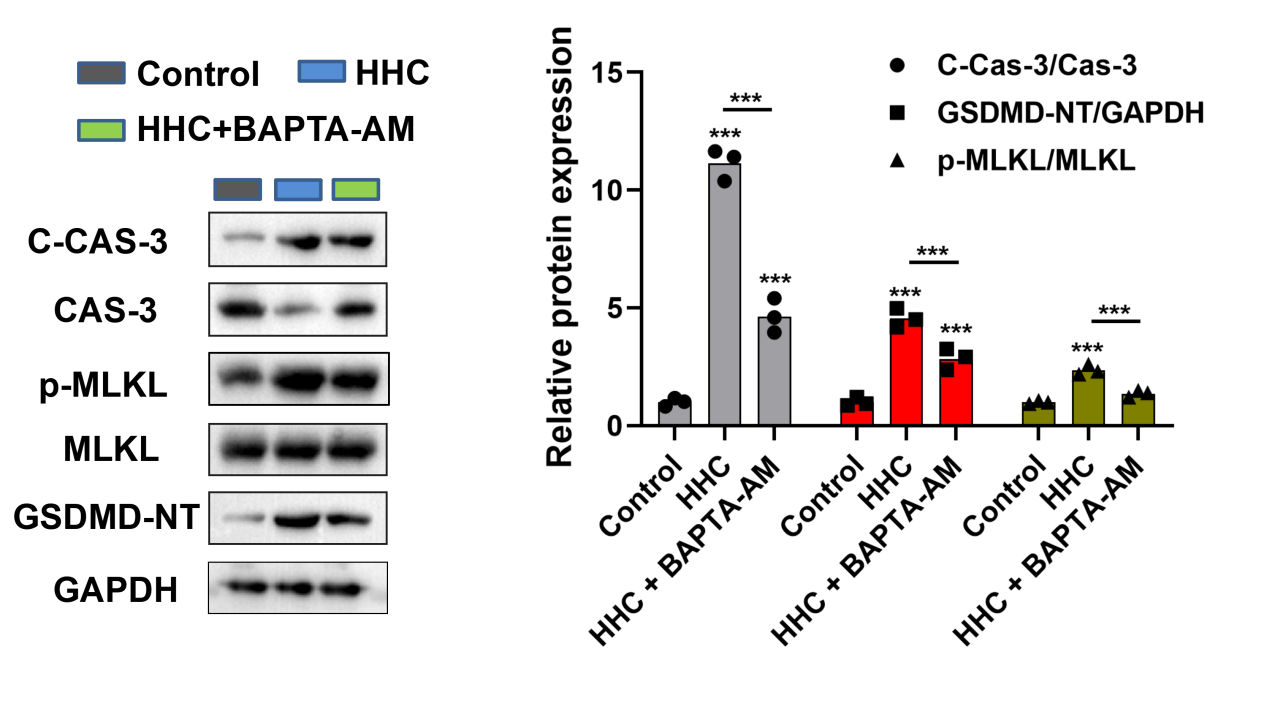


Figure S8. Western blot analysis of key PANoptosis biomarkers, including cleaved caspase-3 (apoptosis), p-MLKL (necroptosis), and GSDMD-NT (pyroptosis) in cells subjected to the indicated treatments. GAPDH was used as the loading control. Data are presented as mean ± SD (n = 3). *******p*** *<* 0.01*, *******p*** *<* 0.001*.*

**
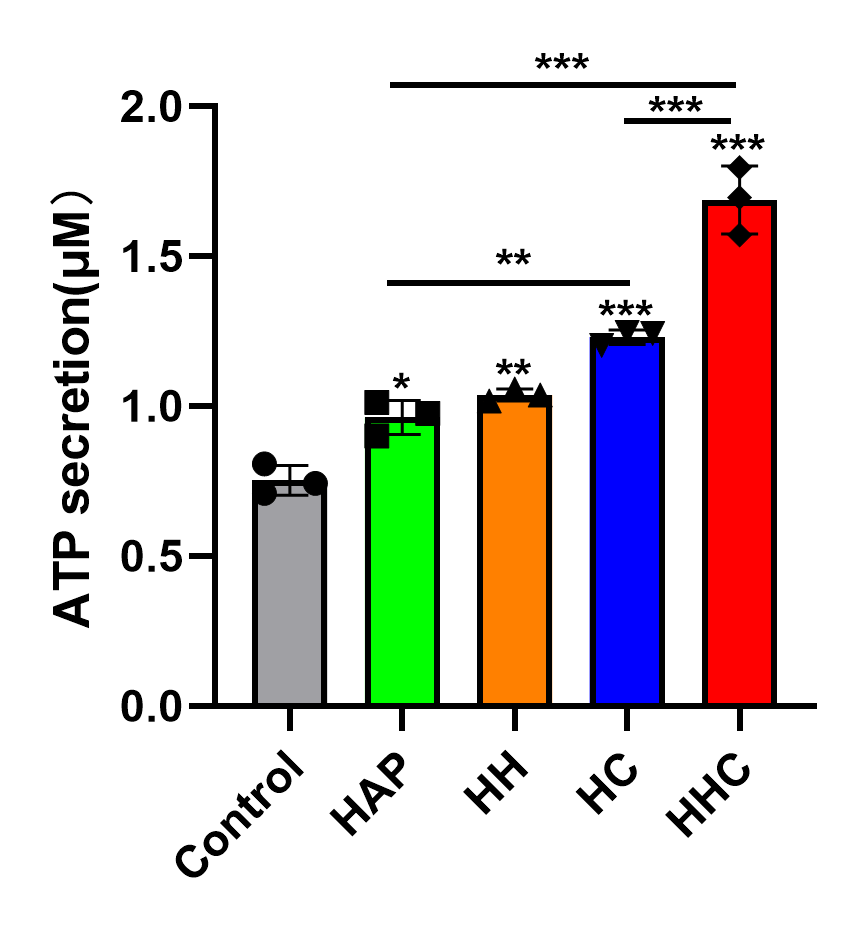
**

Figure S9. ATP secretion in each group. Data are presented as mean ± SD, n = 3. ****p*** < 0.05， *****p*** < 0.01， ******p*** < 0.001.


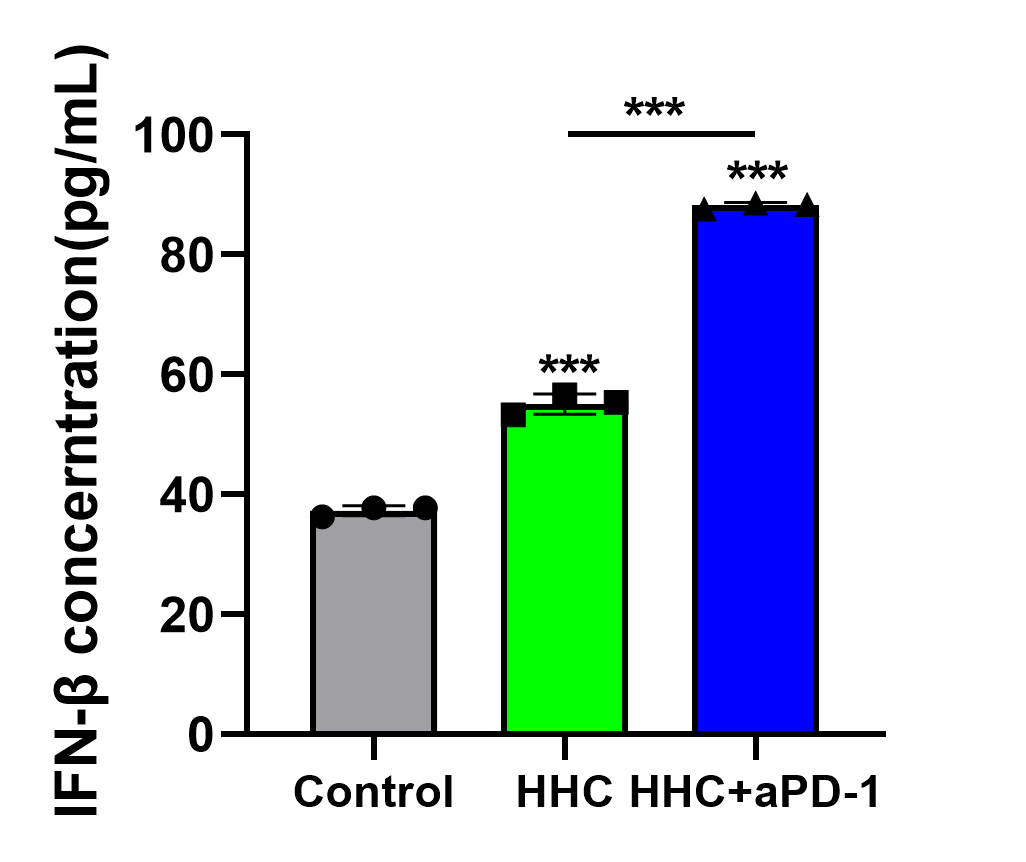


Figure S10. Serum levels of IFN-β in CT26 tumor-bearing mice from different treatment groups, measured by ELISA. Data are presented as mean ± SD. ********p*** < 0.001.


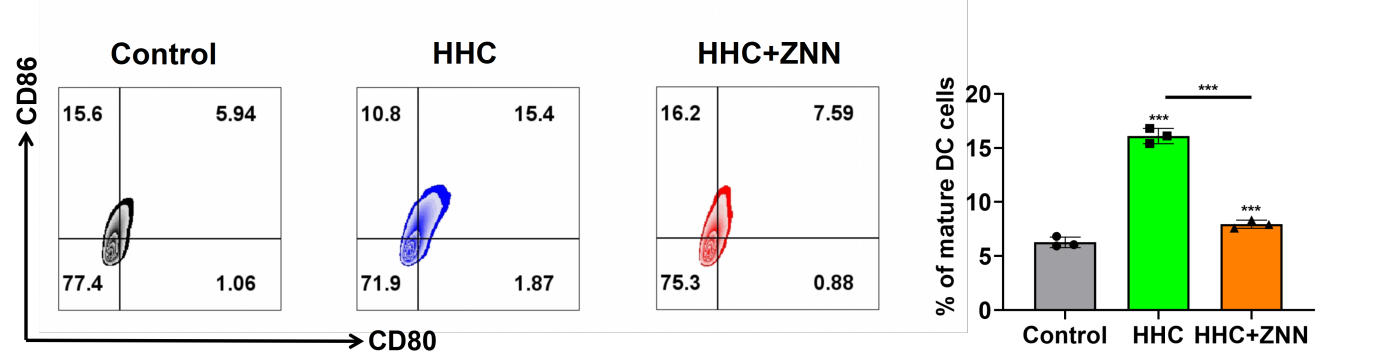


Figure S11. Flow cytometry analysis and quantification of mature bone marrow-derived dendritic cells (BMDCs) (CD80^+^CD86^+^) following co-incubation with the conditioned medium from differently treated CT26 cells. Data are presented as mean ± SD. ********p*** < 0.001.


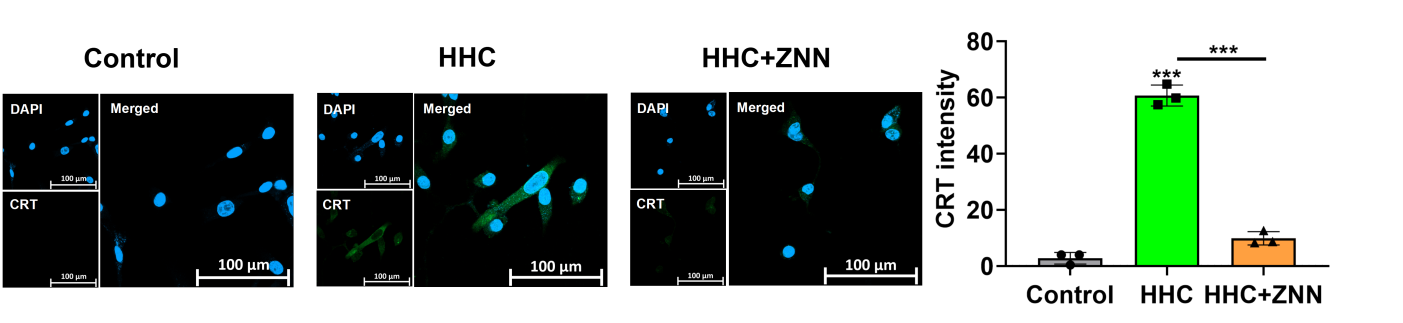


Figure S12. Representative immunofluorescence images showing the surface exposure of calreticulin (CRT) in CT26 cells treated with HHC, with or without the PANoptosis inhibitor cocktail (Z-DEVD-FMK, NLRP3/AIM2-IN-3, and Necrostatin-1). ***** ***p*** < 0.001.


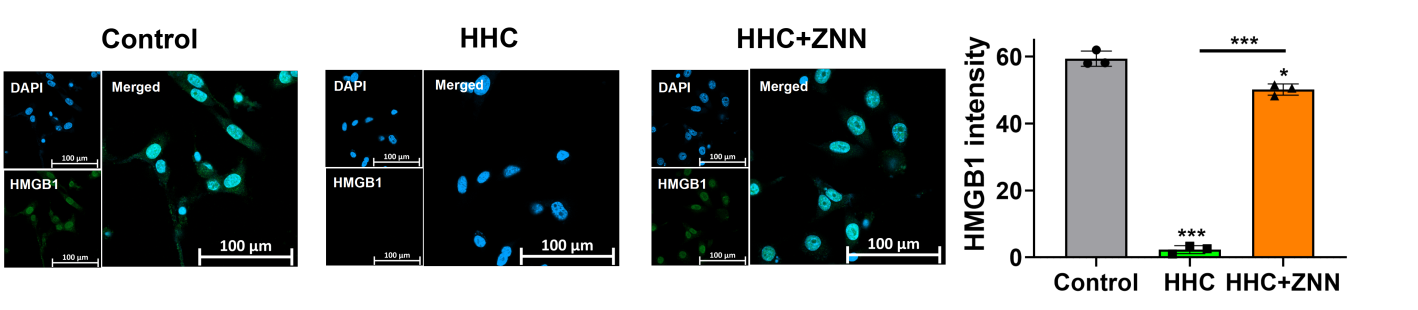


Figure S13. Representative immunofluorescence images showing the nuclear release of HMGB1 in CT26 cells treated with HHC, with or without the PANoptosis inhibitor cocktail (Z-DEVD-FMK, NLRP3/AIM2-IN-3, and Necrostatin-1). ******p*** < 0.05, ********p*** < 0.001.


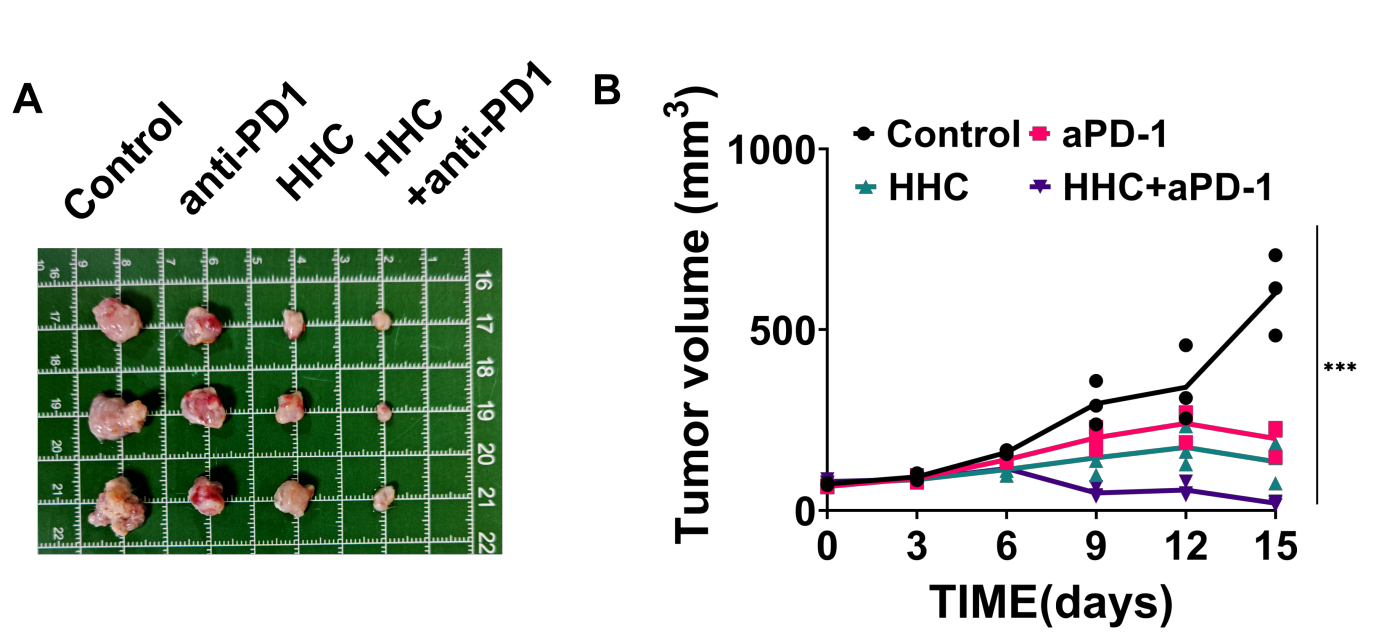


Figure S14. In vivo synergistic antitumor efficacy of HHC combined with anti-PD-1 immune checkpoint blockade. (A) Representative photographic images of excised tumors from CT26 tumor-bearing mice in different treatment groups (Control, anti-PD1, HHC, and HHC + anti-PD-1) at the experimental endpoint. (B) Tumor growth curves of the mice during the 15-day therapeutic period. Data are presented as mean ± SD (n = 3). ********p*** < 0.001.

**
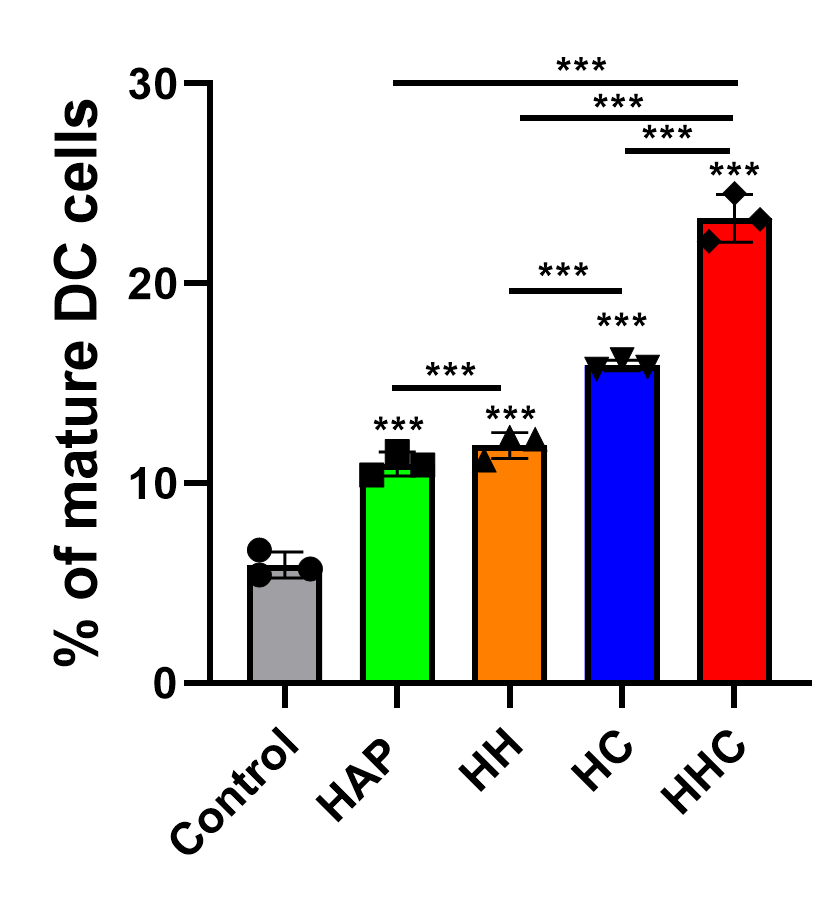
**

Figure S15. Quantitative analysis of dendritic cell (DC) maturation in tumor tissue by flow cytometry. Data are presented as mean ± SD (n = 3). ******p*** < 0.001.

**
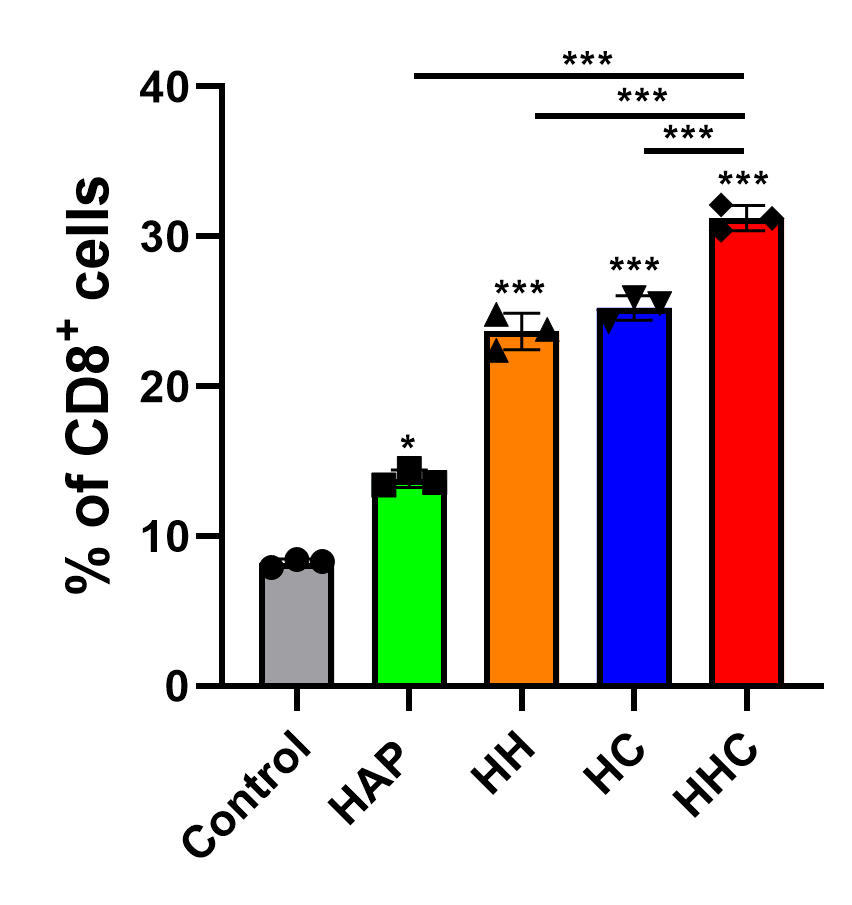
**

Figure S16. Quantitative analysis of CD3^+^CD8^+^ cells (cytotoxic T cells) in tumor tissue by flow cytometry. Data are presented as mean ± SD (n = 5). ****p*** < 0.05， ******p*** < 0.001.


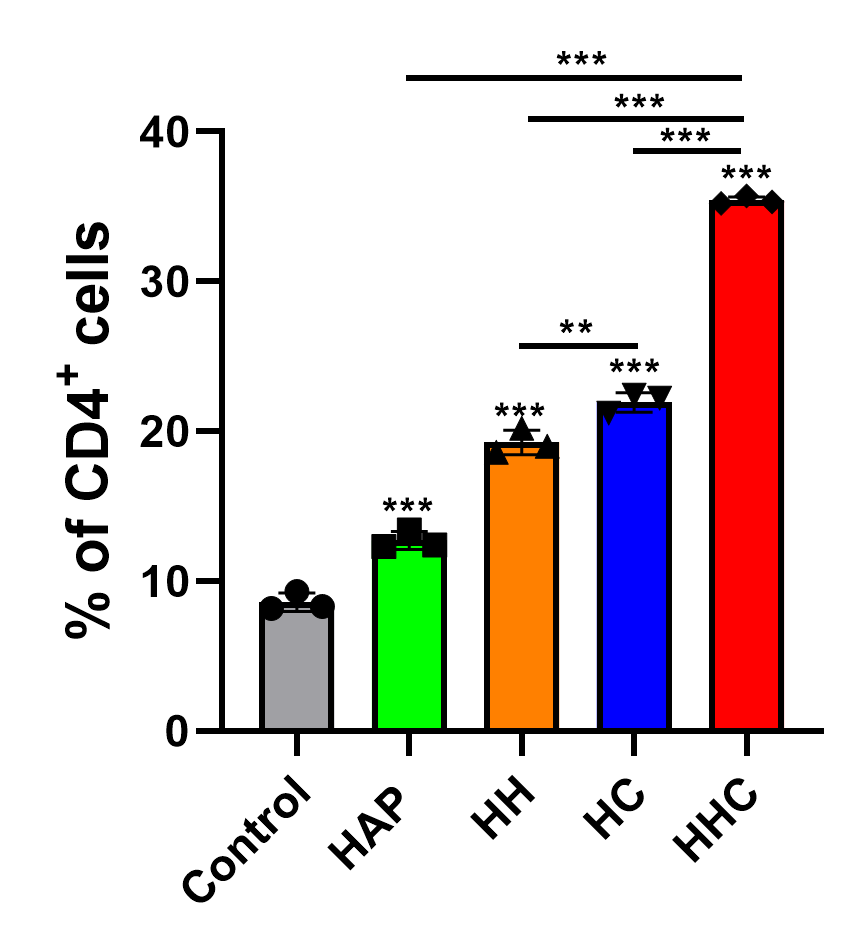


Figure S17. Quantitative analysis of CD3^+^CD4^+^ cells (helper T cells) in tumor tissue by flow cytometry. Data are presented as mean ± SD (n = 5). *****p*** < 0.01， ******p*** < 0.001.


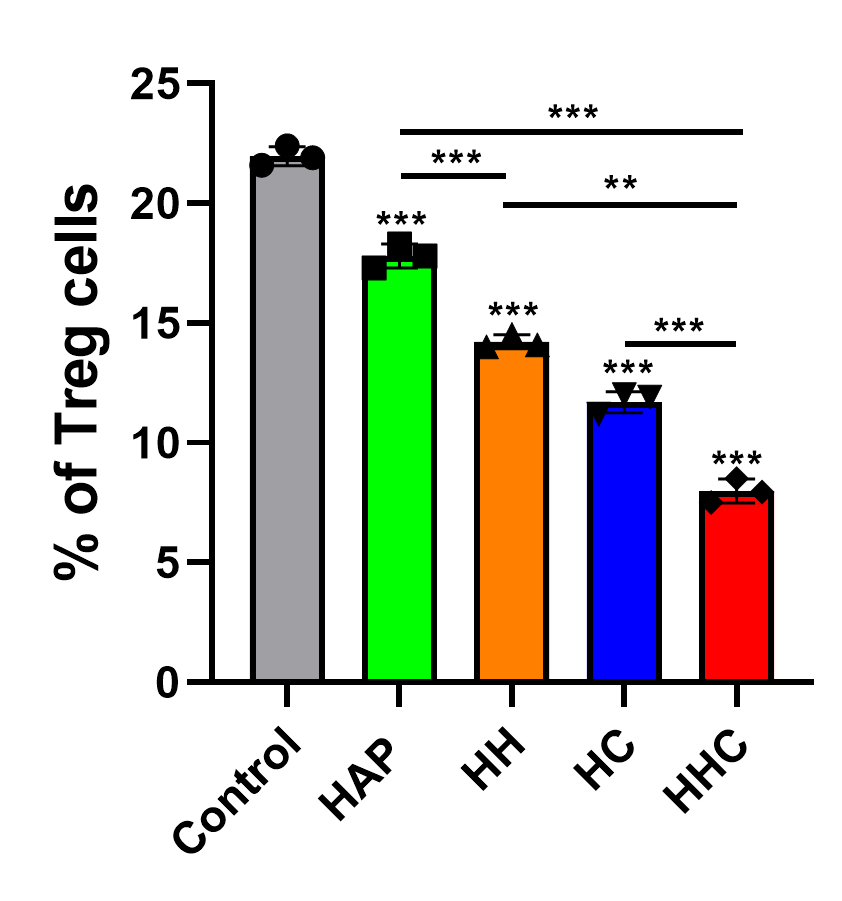


Figure S18. Quantitative analysis of CD4^+^Foxp3^+^ regulatory T cells (Tregs) in tumor tissue by flow cytometry. Data are presented as mean ± SD (n = 5). *****p*** < 0.01， ******p*** < 0.001.


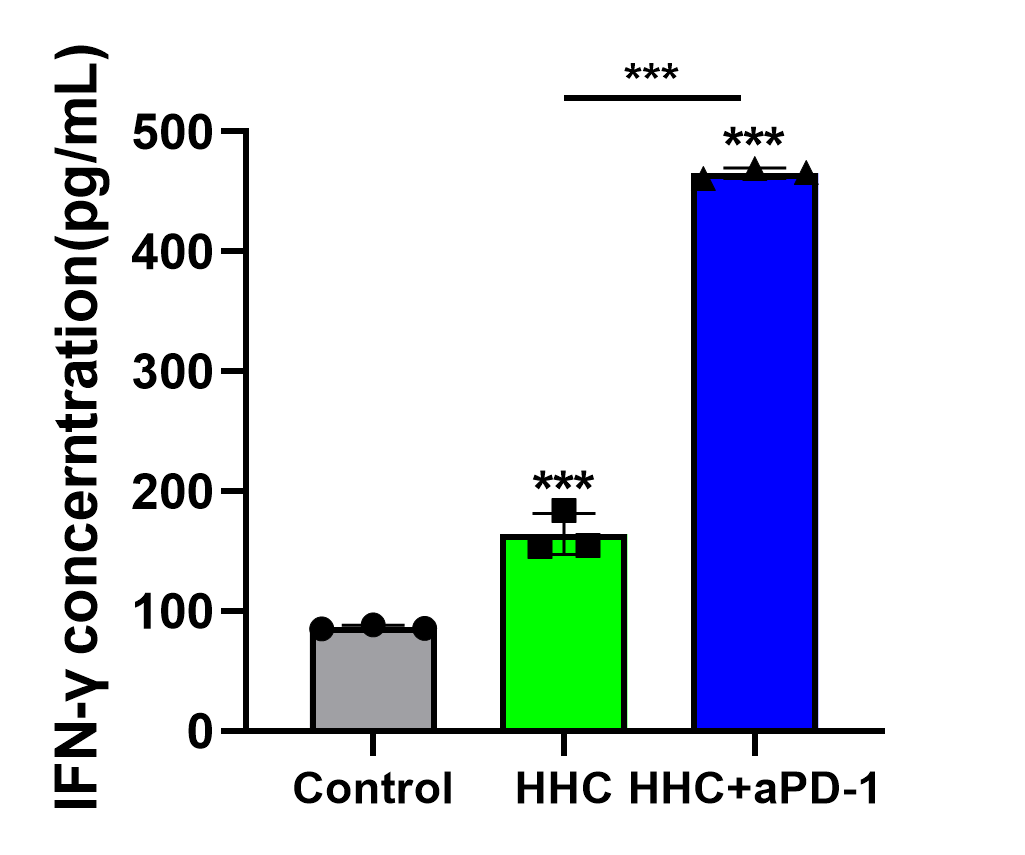


Figure S19. IFN-γ levels in the tumor tissue homogenates of differently treated mice. Data are presented as mean ± SD. ********p*** < 0.001.


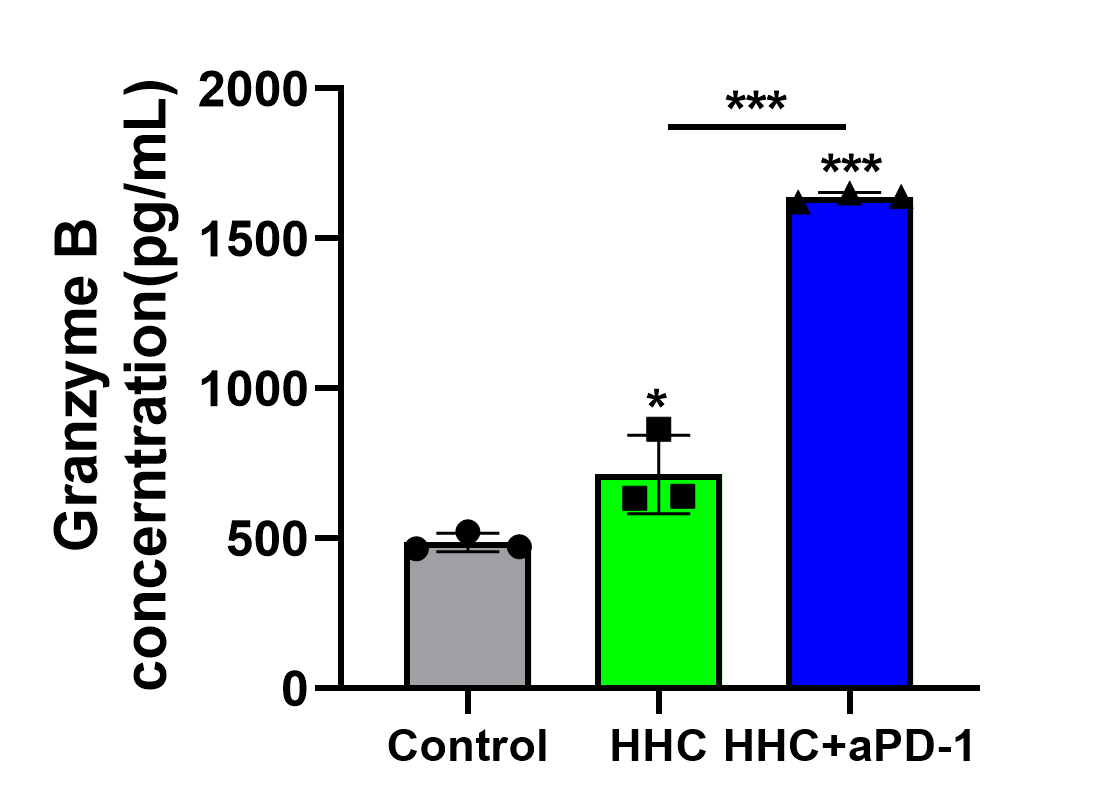


Figure S20. Granzyme B levels in the tumor tissue homogenates of differently treated mice. Data are presented as mean ± SD. ******p*** < 0.05, ********p*** < 0.001.


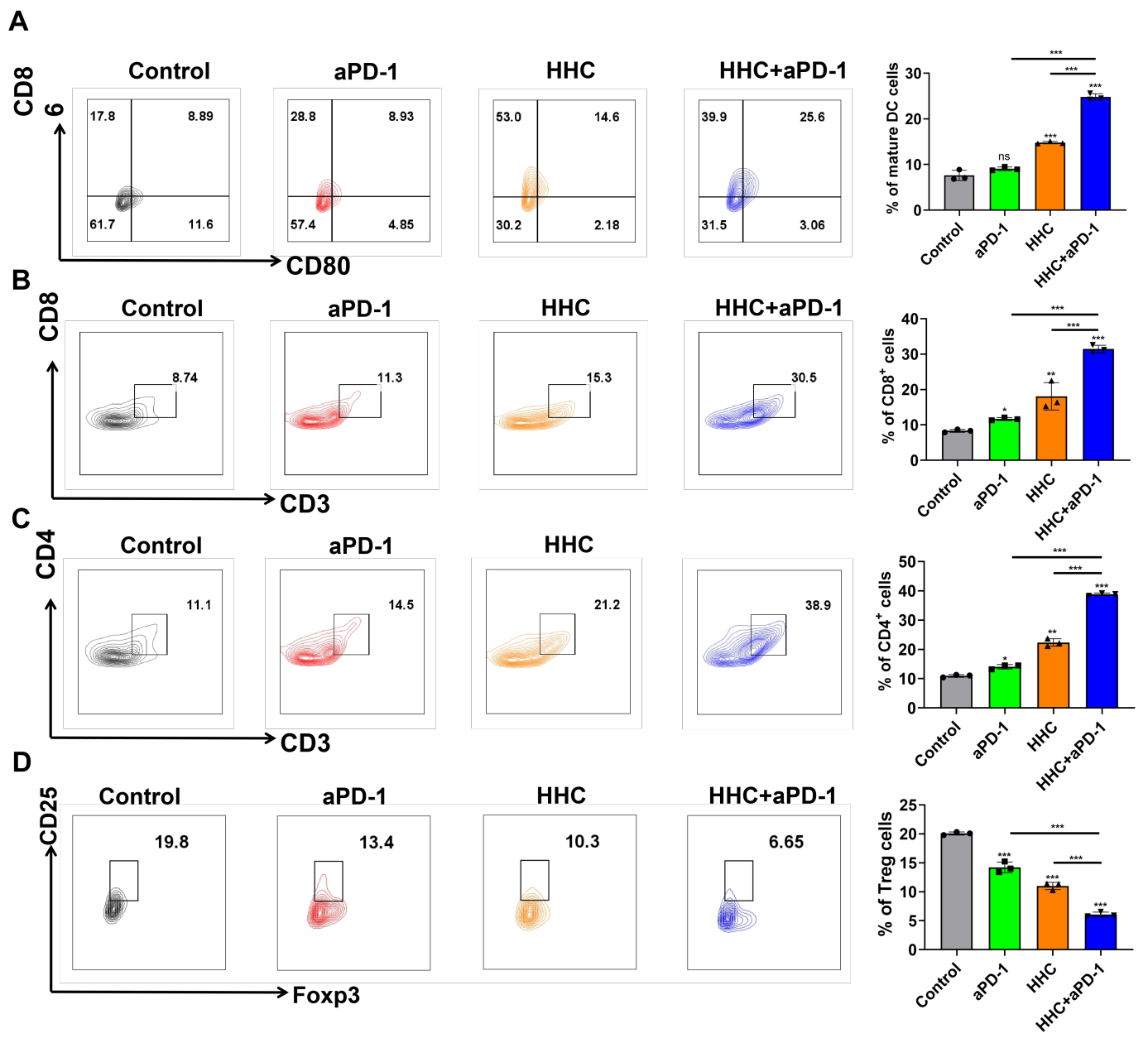


Figure S21. In vivo remodeling of the tumor immune microenvironment by the combination therapy of HHC and anti-PD-1. Representative flow cytometry contour plots (left) and the corresponding quantitative analysis (right) demonstrating the intratumoral populations of specific immune cells in CT26 tumor-bearing mice after various treatments. (A) Relative proportions of mature dendritic cells (mDCs), identified as the CD80^+^CD86^+^ population. (B) Intratumoral infiltration of cytotoxic CD8^+^ T cells (CD3^+^CD8^+^). (C) Intratumoral infiltration of helper CD4^+^ T cells (CD3^+^CD4^+^). (D) Frequency of immunosuppressive regulatory T cells (Tregs), gated as the CD25^+^Foxp3^+^ population. Data are presented as mean ± SD (n = 3). ******p*** < 0.05, *******p*** < 0.01, ********p*** < 0.001.
